# Clinical correlates of CT imaging-derived phenotypes among lean and overweight patients with hepatic steatosis

**DOI:** 10.1038/s41598-023-49470-x

**Published:** 2024-01-02

**Authors:** Isabel Song, Elizabeth W. Thompson, Anurag Verma, Matthew T. MacLean, Jeffrey Duda, Ameena Elahi, Richard Tran, Pavan Raghupathy, Sophia Swago, Mohamad Hazim, Abhijit Bhattaru, Carolin Schneider, Marijana Vujkovic, Drew A. Torigian, Charles E. Kahn, James C. Gee, Arijitt Borthakur, Colleen M. Kripke, Christopher C. Carson, Rotonya Carr, Qasim Jehangir, Yi-An Ko, Harold Litt, Mark Rosen, David A. Mankoff, Mitchell D. Schnall, Haochang Shou, Julio Chirinos, Scott M. Damrauer, Marina Serper, Jinbo Chen, Daniel J. Rader, Penn Medicine BioBank, Marylyn D. Ritchie, JoEllen Weaver, Nawar Naseer, Afiya Poindexter, Khadijah Hu-Sain, Meghan Livingstone, Fred Vadivieso, Stephanie DerOhannessian, Teo Tran, Julia Stephanowski, Monica Zielinski, Ned Haubein, Joseph Dunn, Anurag Verma, Colleen M. Kripke, Marjorie Risman, Renae Judy, Shefali S. Verma, Yuki Bradford, Scott Dudek, Theodore Drivas, Walter R. T. Witschey, Hersh Sagreiya

**Affiliations:** 1 Department of Radiology, Perelman School of Medicine, University of Pennsylvania, 3400 Spruce Street, Philadelphia, PA 19104 USA; 2 Department of Medicine, Perelman School of Medicine, University of Pennsylvania, Philadelphia, PA USA; 3 Department of Genetics, Perelman School of Medicine, University of Pennsylvania, Philadelphia, PA USA; 4 Department of Medicine, School of Medicine, University of Washington, Seattle, WA USA; 5 Department of Biostatistics, Epidemiology, and Informatics, Perelman School of Medicine, University of Pennsylvania, Philadelphia, PA USA; 6 Department of Surgery, Perelman School of Medicine, University of Pennsylvania, Philadelphia, PA USA; 7 Institute for Translational Medicine and Therapeutics, Perelman School of Medicine, University of Pennsylvania, Philadelphia, PA USA; 8 Perelman School of Medicine, University of Pennsylvania, Philadelphia, PA USA; 9 Department of Pathology and Laboratory Medicine, University of Pennsylvania, Philadelphia, PA USA

**Keywords:** Risk factors, Translational research, Hepatology, Medical imaging

## Abstract

The objective of this study is to define CT imaging derived phenotypes for patients with hepatic steatosis, a common metabolic liver condition, and determine its association with patient data from a medical biobank. There is a need to further characterize hepatic steatosis in lean patients, as its epidemiology may differ from that in overweight patients. A deep learning method determined the spleen-hepatic attenuation difference (SHAD) in Hounsfield Units (HU) on abdominal CT scans as a quantitative measure of hepatic steatosis. The patient cohort was stratified by BMI with a threshold of 25 kg/m^2^ and hepatic steatosis with threshold SHAD ≥  − 1 HU or liver mean attenuation ≤ 40 HU. Patient characteristics, diagnoses, and laboratory results representing metabolism and liver function were investigated. A phenome-wide association study (PheWAS) was performed for the statistical interaction between *SHAD* and the binary characteristic *LEAN*. The cohort contained 8914 patients—lean patients with (N = 278, 3.1%) and without (N = 1867, 20.9%) steatosis, and overweight patients with (N = 1863, 20.9%) and without (N = 4906, 55.0%) steatosis. Among all lean patients, those with steatosis had increased rates of cardiovascular disease (41.7 vs 27.8%), hypertension (86.7 vs 49.8%), and type 2 diabetes mellitus (29.1 vs 15.7%) (all *p* < 0.0001). Ten phenotypes were significant in the PheWAS, including chronic kidney disease, renal failure, and cardiovascular disease. Hepatic steatosis was found to be associated with cardiovascular, kidney, and metabolic conditions, separate from overweight BMI.

Nonalcoholic fatty liver disease (NAFLD) is a complex metabolic liver disease whose liver histology ranges from hepatic steatosis to non-alcoholic steatohepatitis with or without fibrosis. In some cases, NAFLD can progress to cirrhosis and even hepatocellular carcinoma^1^. NAFLD is a common though largely asymptomatic condition, affecting approximately 30% of the population worldwide^2^. Although high body mass index (BMI) has been shown to be strongly associated with NAFLD^3^, a subtype termed lean steatosis affects many people in the normal BMI range (between 18.5 and 25 kg/m^2^) and represents approximately 19% of NAFLD patients^4^.

Obesity, hypertension, type 2 diabetes mellitus, hyperlipidemia, and metabolic syndrome are established risk factors for NAFLD^5^. Studies have shown that overweight patients with hepatic steatosis tend to exhibit these characteristics to a greater degree compared to lean patients with steatosis^6^. Others have demonstrated an association between NAFLD and both cardiovascular disease (CVD) and chronic kidney disease (CKD), indicating that these associations hold independent of obesity^7–9^. Though there are many factors that may contribute to this relationship, elevated levels of triglycerides, along with other components of metabolic syndrome associated with NAFLD, have been shown to increase the risk of CVD^7^. Though rates of cirrhosis, cardiovascular complications, and other comorbid conditions for lean NAFLD patients have been found to be lower than for overweight NAFLD patients^10^, studies have shown that even lean NAFLD is associated with metabolic syndrome and insulin resistance. However, the prevalence of lean NAFLD, its associated clinical characteristics, and its outcomes are not well characterized^1^. There may also be racial differences in the incidence of metabolic diseases with lean NAFLD, with members of Asian populations being more likely to develop insulin resistance and type 2 diabetes mellitus at lower BMI levels^11^. Genetics also plays a factor in hepatic steatosis risk, as being a homozygous carrier of the PNPLA3 rs738409 variant has been found to increase the risk for hepatic steatosis by more than double^5^.

Liver biopsy remains the reference standard method to measure hepatic fat deposition and to diagnose hepatic steatosis. However, liver biopsy is invasive and is therefore unlikely to be recommended for patients who do not already show other signs of liver damage. In contrast, computed tomography (CT) imaging is widely accessible to patients undergoing evaluation for all manner of medical disorders, frequently uncovering incidental findings. With over 70 million CT scans performed annually in the United States alone, there is an abundance of CT studies available for analysis^12^. It is possible to reliably quantify hepatic steatosis by comparing liver attenuation to spleen attenuation on CT. Lower liver attenuation compared to the spleen attenuation typically indicates a greater degree of hepatic steatosis, as the relatively lower density of fat compared with other tissues causes a decrease in attenuation^13^. Moderate to severe hepatic steatosis is formally diagnosed when the attenuation of the liver on unenhanced CT is at least 10 Hounsfield units (HU) less than the attenuation of the spleen or if the attenuation of the liver is less than 40 HU^14^. A spleen-liver attenuation difference threshold of  − 1 has been proposed for including all significant degrees of hepatic steatosis (> 5% steatosis of the liver)^15^.

With the recent popularization of machine learning techniques, artificial intelligence (AI) has shown great potential in efficiently processing large amounts of data. Some studies have used automated means, such as convolutional neural networks (CNNs), to successfully quantify hepatic fat and diagnose hepatic steatosis^16–18^. The combination of AI and commonly performed non-invasive imaging methods such as CT and magnetic resonance imaging (MRI) can help to efficiently identify patients who have hepatic steatosis.

For this study, deep learning methods were used to identify patients from the Penn Medicine BioBank with hepatic steatosis. In combination with other electronic medical record data from the biobank, we aimed to establish associations between hepatic steatosis and other phenotypes in lean patients and to compare the clinical laboratory values of lean and overweight patients with and without steatosis. The objective of this study is to identify the prevalence and clinical characteristics associated with lean NAFLD in a large, diverse health system biobank.

## Results

### Cohort analysis

**Figure 1:**
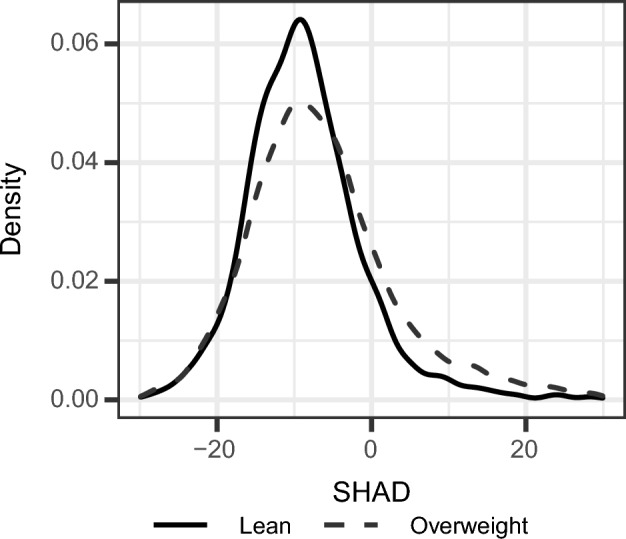
Density of SHAD values for lean and overweight patients. *p* < 0.0001 as determined by the Wilcoxon test. Lean patients have 18.5 kg/m^2^ ≤ BMI < 25 kg/m^2^. Patients with steatosis have SHAD ≥  − 1 HU or LMA < 40 HU. *BMI* body mass index, *SHAD* spleen-hepatic attenuation difference, *LMA* liver mean attenuation.

We report patient characteristics stratified by the four groups based on BMI category and hepatic steatosis in Table 1. Hepatic steatosis was quantified by the spleen-hepatic attenuation difference as measured from patient CT scans, with a threshold of SHAD ≥ -1 HU^15^ or LMA < 40 HU^14^. In both the lean and overweight groups, a larger proportion of patients with steatosis crossed the SHAD ≥ -1 HU threshold than the LMA < 40 HU threshold, with 97% versus 37% for lean patients and 82% versus 64% for overweight patients. In other words, fewer patients were identified as having hepatic steatosis based on low liver attenuation rather than a high SHAD. Of the 8914 patients in the cohort, 24.0% had hepatic steatosis (N = 2141) and 24.1% had a lean BMI (N = 2145). A total of 13.0% of patients with hepatic steatosis were lean (N = 278), while 27.6% of patients without hepatic steatosis were lean (N = 1867). The distributions of SHAD values for lean and overweight patients in the cohort are shown in Fig. 1. Lean patients had significantly lower SHAD values overall (median =  − 9.29 HU, IQR = 8.63 HU) compared to overweight patients (median =  − 7.62 HU, IQR = 10.94 HU), indicating a lower liver fat content (*p* < 0.0001).

**Table 1:** Demographic and clinical characteristics of the patient cohort used in the study.

	Total (N = 8914)	Lean (18.5 kg/m^2^ ≤ BMI < 25 kg/m^2^)		Overweight (BMI ≥ 25 kg/m^2^)		*p*-Value
		With steatosis (N = 278)	Without steatosis (N = 1867)	With steatosis (N = 1863)	Without steatosis (N = 4906)	
Age (years)	63 [19]	65.5 [20.75]	62 [21]	62 [16]	63 [18]	0.013
Sex (n (%))						
Male	4895 (54.9%)	149 (53.6%)	849 (45.5%)	1,161 (62.3%)	2,736 (55.8%)	< 0.0001
Female	4019 (45.1%)	129 (46.4%)	1,018 (54.5%)	702 (37.7%)	2,170 (44.2%)	
Race (n (%))						
White	6396 (71.8%)	193 (69.4%)	1,446 (77.5%)	1,378 (74.0%)	3,379 (68.9%)	< 0.0001
Black	2031 (22.8%)	59 (21.2%)	297 (15.9%)	394 (21.1%)	1,281 (26.1%)	
Asian	144 (1.6%)	8 (2.9%)	51 (2.7%)	24 (1.3%)	61 (1.2%)	
Other/Unknown	343 (3.8%)	18 (6.5%)	73 (3.9%)	67 (3.6%)	185 (3.8%)	
BMI (kg/m^2^)	28 [8]	23 [3]	22 [2.5]	32 [8]	29 [6]	–
LMA (HU)	51.50 [12.30]	42.43 [10.19]	55.86 [8.63]	37.78 [11.65]	53.21 [9.53]	–
SHAD (HU)	− 8.09 [10.32]	2.44 [7.15]	− 10.16 [7.27]	2.91 [9.32]	− 10.15 [7.97]	–
CVD (n (%))	3001 (33.7%)	116 (41.7%)	519 (27.8%)	697 (37.4%)	1,669 (34.0%)	< 0.0001
HTN (n (%))	5676 (63.7%)	191 (68.7%)	930 (49.8%)	1,371 (73.6%)	3,184 (64.9%)	< 0.0001
T2DM (n (%))	2482 (27.8%)	81 (29.1%)	293 (15.7%)	793 (42.6%)	1,315 (26.8%)	< 0.0001
HLD (n (%))	5120 (57.4%)	160 (57.6%)	871 (46.7%)	1,187 (63.7%)	2,902 (59.2%)	< 0.0001

Age, BMI, LMA, and SHAD values listed for each group are the in the format of median [interquartile range]. A higher value for SHAD corresponds to increased hepatic steatosis. *p*-Values were calculated with the Kruskal–Wallis test for continuous fields (age) and chi-square for categorical fields (sex, race, CVD, HTN, T2DM, HLD) and were corrected using the Benjamini–Hochberg method for a significance threshold of *p* < 0.05. *p*-Values were not calculated for BMI, LMA, and SHAD because the groups were dichotomized by these quantities. Lean patients have 18.5 kg/m^2^ ≤ BMI < 25 kg/m^2^. Patients with steatosis have SHAD ≥  − 1 HU or LMA < 40 HU.

*BMI* body mass index, *LMA* liver mean attenuation, *SHAD* spleen-hepatic attenuation difference, *CVD* cardiovascular disease, *HTN* hypertension, *T2DM* type 2 diabetes mellitus, *HLD *hyperlipidemia.

Chi-squared and p-values from post-hoc chi-square testing for the categorical demographic and clinical characteristics are detailed in Table 2. Nominal, uncorrected p-values are listed in Supplementary Table S1 for reference. Rates of the clinical conditions—CVD, HTN, T2DM, and HLD—differed between the patient groups. Among lean patients, those with hepatic steatosis had significantly higher rates of CVD (41.7 vs 27.8%, *p* < 0.0001), HTN (68.7 vs 49.8%, *p* < 0.0001), T2DM (29.1 vs 15.7%, *p* < 0.0001) and HLD (57.6 vs 46.7%, *p* = 2.1E−3). Additionally, among patients with hepatic steatosis, rates of HTN (68.7 vs 73.6%, *p* = 0.031), T2DM (29.1 vs 42.6%, *p* < 0.0001), and HLD (57.6 vs 53.7%, *p* = 9.1E−3) varied significantly depending on whether they were lean or overweight. We also found that lean patients with steatosis had comparable rates of clinical conditions to overweight patients without steatosis (*p* ≥ 0.20) apart from a higher rate of CVD (41.7 vs 34.0%, *p* = 9.1E−3).

**Table 2:** Chi-squared and p-values from pairwise chi-square testing for clinical and demographic categorical characteristics.

	Lean with steatosis vs Lean without steatosis		Lean with steatosis vs Overweight with steatosis		Lean without steatosis vs Overweight with steatosis		Lean with steatosis vs Overweight without steatosis		Lean without steatosis vs Overweight without steatosis		Overweight with steatosis vs Overweight without steatosis	
	χ^2^	*p*-Value	χ^2^	*p*-Value	χ^2^	*p*-Value	χ^2^	*p*-Value	χ^2^	*p*-Value	χ^2^	*p*-Value
Sex	6.1	**0.017**	7.39	**9.1E-3**	105.8	** < 0.0001**	0.42	0.53	57.11	** < 0.0001**	23.45	** < 0.0001**
Race	9.93	**0.024**	9.82	**0.024**	25.23	** < 0.0001**	12.63	**8.6E-3**	92.57	** < 0.0001**	18.55	**5.8E-4**
CVD	23.31	** < 0.0001**	2.08	0.17	39.79	** < 0.0001**	7.39	**9.1E-3**	24.02	** < 0.0001**	7.01	**0.011**
HTN	38.26	** < 0.0001**	4.93	**0.031**	280.6	** < 0.0001**	0.97	0.34	174.44	** < 0.0001**	54.16	** < 0.0001**
T2DM	27.35	** < 0.0001**	24.21	** < 0.0001**	354.8	** < 0.0001**	0.08	0.78	105.24	** < 0.0001**	159.48	** < 0.0001**
HLD	10.4	**2.1E-3**	7.45	**9.1E-3**	135.66	** < 0.0001**	1.12	0.32	107.41	** < 0.0001**	14.58	**2.4E-4**

*p*-Values were adjusted with the Benjamini–Hochberg method for a *p* < 0.05 level of statistical significance. Lean patients have 18.5 kg/m^2^ ≤ BMI < 25 kg/m^2^. Patients with steatosis have SHAD ≥  − 1 HU or LMA < 40 HU.

*BMI* body mass index, *SHAD* spleen-hepatic attenuation difference, *LMA* liver mean attenuation, *CVD* cardiovascular disease, *HTN* hypertension, *T2DM* type 2 diabetes mellitus, *HLD* hyperlipidemia.

Significant values are bold.

Hepatic steatosis rates differed across race and sex in both BMI categories. Lean Black patients were significantly more likely to have hepatic steatosis than lean White patients, but the opposite was true among the overweight group, with overweight White patients being more likely to have hepatic steatosis than overweight Black patients. Male patients were significantly more likely to have steatosis than female patients among both overweight (29.8 vs 24.4%, p < 0.0001) and lean patients (14.9 vs 11.2%, *p* = 0.014).

In examining the genetic information available among patients in the cohort, we observed that for both lean patients and overweight patients, homozygous carriers of the PNPLA3 rs738409 variant were more prevalent among those with steatosis than among those without steatosis. This difference was statistically significant among both lean patients (10.3 vs 4.6%, *p* = 1.9E−3) and overweight patients (7.7 vs 3.7%, *p* < 0.0001). The difference between lean and overweight patients with steatosis was not statistically significant (10.3 vs 7.7%, *p* = 0.28). Results are summarized in Table 3.

**Table 3:** Number of carriers of the PNPLA3 I148M variant.

	Lean with steatosis (N = 195)	Lean without steatosis (N = 1383)	Overweight with steatosis (N = 1410)	Overweight without steatosis (N = 3778)
Non-carrier	112 (57.4%)	867 (62.7%)	779 (55.2%)	2461 (65.1%)
Heterozygous	63 (32.3%)	452 (32.7%)	522 (37.0%)	1179 (31.2%)
Homozygous	20 (10.3%)	64 (4.6%)	109 (7.7%)	138 (3.7%)

Lean patients have 18.5 kg/m^2^ ≤ BMI < 25 kg/m^2^. Patients with steatosis have SHAD ≥  − 1 HU or LMA < 40 HU. The rate of patients who are homozygous carriers by the chi-squared test: *p* = 1.9E−3 for lean patients with vs. without steatosis, *p* < 0.0001 for overweight patients with vs. without steatosis, and *p* = 0.28 for lean vs. overweight patients with steatosis.

Using the moderate-to-severe steatosis definition of SHAD ≥ 10 HU or LMA < 40 HU, associations were similar (Supplementary Tables  S2, S3, S4).

Performing the same analyses of demographic and clinical characteristics with the patient cohort in three BMI categories (lean, overweight, obese), we found that obese patients with steatosis had significantly greater SHAD values, indicating more severe degrees of steatosis, as well as higher rates of HTN and T2DM than both lean and overweight patients with steatosis. However, overweight patients with steatosis had the highest rates of HLD while lean patients with steatosis had the highest rates of CVD, though neither reached the threshold for statistical significance (both *p* > 0.1). The results are shown in Supplementary Tables S5, S6; Fig. S1.

### Blood biomarker analysis

**Figure 2:**
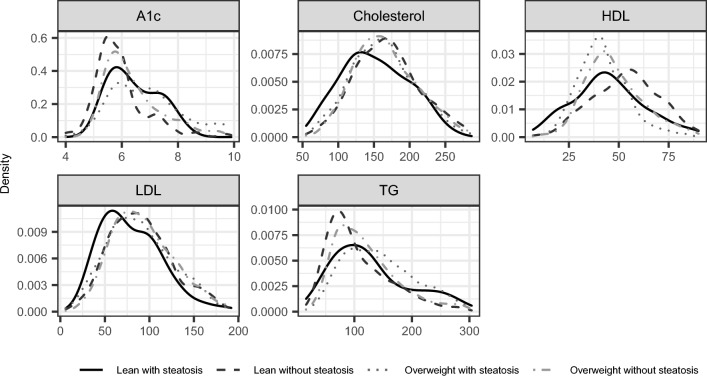
Metabolic blood biomarker distributions of the patient cohorts. Lean patients have 18.5 kg/m^2^ ≤ BMI < 25 kg/m^2^. Patients with steatosis have SHAD ≥  − 1 HU or LMA < 40 HU. *BMI* body mass index, *SHAD* spleen-hepatic attenuation difference, *LMA* liver mean attenuation, *A1c* hemoglobin A1c, *HDL* high-density lipoprotein, *LDL* low-density lipoprotein, *TG*  triglycerides.

**Figure 3:**
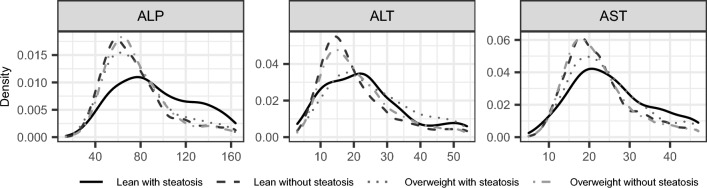
Liver function biomarker distributions of the patient groups. Lean patients have 18.5 kg/m^2^ ≤ BMI < 25 kg/m^2^. Patients with steatosis have SHAD ≥  − 1 HU or LMA < 40 HU. *BMI* body mass index, *SHAD* spleen-hepatic attenuation difference, *LMA* liver mean attenuation, *ALP* alkaline phosphatase, *ALT* alanine transaminase, *AST* aspartate transaminase.

Density plots of the metabolic biomarker values in each group and their liver enzymes are illustrated in Figs. 2 and 3 respectively, and group means and p-values by the Kruskal–Wallis test are detailed in Table 4. Z-scores and *p*-values from Dunn’s test for multiple comparisons are detailed in Table 5. Nominal, uncorrected p-values are included for reference in Supplementary Table S7.

**Table 4:** Biomarker median values and number of samples over the patient cohort.

	Lean with steatosis		Lean without steatosis		Overweight with steatosis		Overweight without steatosis		*p*-Value
	N	Median [IQR]	N	Median [IQR]	N	Median [IQR]	N	Median [IQR]	
A1c (%)	18	6.2 [1.625]	102	5.7 [0.8]	174	6.9 [1.6]	368	6.1 [1.3]	< 0.0001
Cholesterol (mg/dL)	83	151 [66.5]	514	167 [59.75]	533	161 [59]	1451	162 [61]	0.013
HDL (mg/dL)	70	43.5 [22]	460	54 [22]	499	41 [15.5]	1367	45 [19]	< 0.0001
LDL (mg/dL)	74	73 [48.25]	484	88 [48]	484	87 [51]	1360	89 [49]	2.8E−3
TG (mg/dL)	94	114 [91.5]	572	90 [78.25]	595	137 [91]	1548	109 [72]	< 0.0001
ALP (U/L)	195	88 [54]	1119	69 [33]	1138	72 [38]	2707	70 [33]	< 0.0001
ALT (U/L)	217	22 [16]	1267	17 [12]	1251	23 [17]	3143	19 [14]	< 0.0001
AST (U/L)	205	24 [14]	1250	21 [9]	1247	22 [11.5]	3092	20 [10]	< 0.0001

*p*-Values were corrected using the Benjamini–Hochberg method for a threshold of *p* < 0.05 for statistical significance. Lean patients have 18.5 kg/m^2^ ≤ BMI < 25 kg/m^2^. Patients with steatosis have SHAD ≥ -1 HU or LMA < 40 HU.

*BMI* body mass index, *SHAD* spleen-hepatic attenuation difference, *LMA* liver mean attenuation, *IQR* interquartile range, *A1c* hemoglobin A1c, *ALP* alkaline phosphatase, *ALT* alanine transaminase, *AST* aspartate transaminase, *HDL* high-density lipoprotein, *LDL* low-density lipoprotein, *TG* triglycerides.

**Table 5:** Z-scores and p-values from Dunn’s test for multiple comparisons for biomarkers that showed a difference between the four groups of the patient cohort by the Kruskal–Wallis test.

	Lean with steatosis vs Lean without steatosis		Lean with steatosis vs Overweight with steatosis		Lean without steatosis vs Overweight with steatosis		Lean with steatosis vs Overweight without steatosis		Lean without steatosis vs Overweight without steatosis		Overweight with steatosis vs Overweight without steatosis	
	Z	*p*	Z	*p*	Z	*p*	Z	*p*	Z	*p*	Z	*p*
A1c (%)	2.29	**0.035**	− 1.23	0.24	− 7.14	** < 0.0001**	0.47	0.64	− 4.2	** < 0.0001**	4.56	** < 0.0001**
Cholesterol (mg/dL)	− 2.97	**6.0E − 3**	− 1.91	0.076	2.02	0.061	− 2.55	**0.018**	1.22	0.24	− 1.23	0.24
HDL (mg/dL)	− 4.23	** < 0.0001**	1.77	0.10	11.88	** < 0.0001**	− 1.22	0.24	7.29	** < 0.0001**	− 7.17	** < 0.0001**
LDL (mg/dL)	− 2.63	**0.015**	− 2.28	**0.035**	0.68	0.51	− 3.32	**1.94E − 3**	− 1.3	0.23	− 2.12	**0.049**
TG (mg/dL)	3.27	**2.3E − 3**	− 2.86	**8.2E − 3**	− 11.62	** < 0.0001**	0.82	0.44	− 5.65	** < 0.0001**	8.38	** < 0.0001**
ALP (U/L)	7.32	** < 0.0001**	5.82	** < 0.0001**	− 2.78	**0.010**	6.87	** < 0.0001**	− 1.67	0.12	1.63	0.13
ALT (U/L)	4.16	** < 0.0001**	− 2.27	**0.035**	− 11.85	** < 0.0001**	2.45	**0.023**	− 4.02	**1.3E − 4**	10.13	** < 0.0001**
AST (U/L)	4.69	** < 0.0001**	1.87	0.082	− 5.31	** < 0.0001**	5.25	** < 0.0001**	0.75	0.47	7.09	** < 0.0001**

*p*-Values were corrected using Benjamini–Hochberg method for a threshold of *p* < 0.05 for statistical significance. Lean patients have 18.5 kg/m^2^ ≤ BMI < 25 kg/m^2^. Patients with steatosis have SHAD ≥  − 1 HU or LMA < 40 HU.

*BMI* body mass index, *SHAD* spleen-hepatic attenuation difference, *LMA* liver mean attenuation, *A1c* hemoglobin A1c, *ALP* alkaline phosphatase, *ALT* alanine transaminase, *AST* aspartate transaminase, *HDL* high-density lipoprotein, *LDL* low-density lipoprotein, *TG* triglycerides.

Significant values are bold.

Of the liver function tests, lean patients with steatosis had significantly higher levels of ALP (median = 88 U/L, IQR = 54 U/L) and AST (median = 24 U/L, IQR = 14 U/L) than both lean patients without steatosis (ALP: median = 69 U/L, IQR = 33 U/L; AST: median = 21 U/L, IQR = 9 U/L) and overweight patients without steatosis (ALP: median = 70 U/L, IQR = 33 U/L; AST: median = 20 U/L, IQR = 10 U/L) (all *p* < 0.001). Lean patients with steatosis also had higher ALT compared to lean patients without steatosis (median = 22 U/L, IQR = 16 U/L vs. median = 17 U/L, IQR = 12 U/L; *p* < 0.0001). Lean patients with steatosis also had significantly higher ALP levels than overweight patients with steatosis (median = 88 U/L, IQR = 54 U/L vs. median = 72 U/L, IQR = 38 U/L; *p* < 0.0001), but comparable ALT and AST levels (*p* ≥ 0.12).

Among the metabolic tests, lean patients with steatosis had significantly lower HDL levels than their counterparts without steatosis (median = 43.5 mg/dL, IQR = 22 mg/dL vs. median = 54 U/L, IQR = 22 mg/dL; *p* < 0.0001). This relationship held for overweight patients as well, with the addition of higher A1c and TG levels among patients with steatosis (*p* < 0.0001). Lean patients with steatosis had the lowest LDL levels among four groups (median = 73 mg/dL, IQR = 48.25 mg/dL, all *p* ≤ 0.035). Lean patients with steatosis and overweight patients without steatosis had comparable levels of A1c, HDL, and TG (all *p* ≥ 0.24). Compared to overweight patients with steatosis, lean patients with steatosis had slightly lower A1c, LDL, and TG, as well as higher HDL, but the results were not statistically significant. After repeating the analyses for the moderate-to-severe steatosis definition of SHAD ≥ 10 or LMA < 40, associations were similar (Supplementary  Figs. S2 and S3; Tables S8 and S9).

The FIB-4 index was used to evaluate the severity of fibrosis of the liver for patients with steatosis, with a score of FIB-4 ≥ 3.25 indicating severe fibrosis^19^. A comparison between the FIB-4 scores of lean patients with steatosis and overweight patients with steatosis is summarized in Table 6. The proportion of lean patients with steatosis who also had severe fibrosis was significantly higher than for overweight patients with steatosis (14.5 vs 8.2%, *p* = 0.038). However, the distribution of FIB-4 scores themselves did not reach the threshold for significance (*p* = 0.36).

**Table 6:** FIB-4 scores for lean and overweight patients with steatosis.

	Lean with steatosis	Overweight with steatosis	*p*-Value
N	124	683	–
Severe fibrosis (n (%))	18 (14.5%)	56 (8.2%)	0.038
Median [IQR]	1.52 [1.24]	1.39 [1.03]	0.36

*p*-Values were calculated using the chi-square test and the Wilcoxon test for the number of patients with severe fibrosis and FIB-4 distribution, respectively. A FIB-4 score of FIB-4 ≥ 3.25 was considered to indicate severe fibrosis. Lean patients have 18.5 kg/m^2^ ≤ BMI < 25 kg/m^2^. Patients with steatosis have SHAD ≥  − 1 HU or LMA < 40 HU

*BMI* body mass index, *SHAD* spleen-hepatic attenuation difference, *LMA* liver mean attenuation.

Performing the same analyses with the patient cohort in three BMI categories (lean, overweight, obese), we found that lean patients with steatosis had significantly lower levels of LDL (*p* = 0.043), TG (*p* = 3.2E−3), and ALT (*p* = 0.036), and significantly higher levels of HDL (*p* = 0.029) and ALP (*p* < 0.0001) than obese patients with steatosis. Overweight patients with steatosis were more similar in biomarker profile to obese patients with steatosis, having only significantly higher HDL (*p* = 4.9E−3). In comparing the degree of fibrosis using the FIB-4 index, both lean and overweight patients with steatosis had a significantly higher proportion of patients with severe fibrosis, compared with obese patients with steatosis (*p* = 1.2E−3 and 2.5E−4). The results with the cohort in three BMI categories are shown in Supplementary Figs. S4 and S5; Tables S10, S11, S12.

### PheWAS

**Figure 4:**
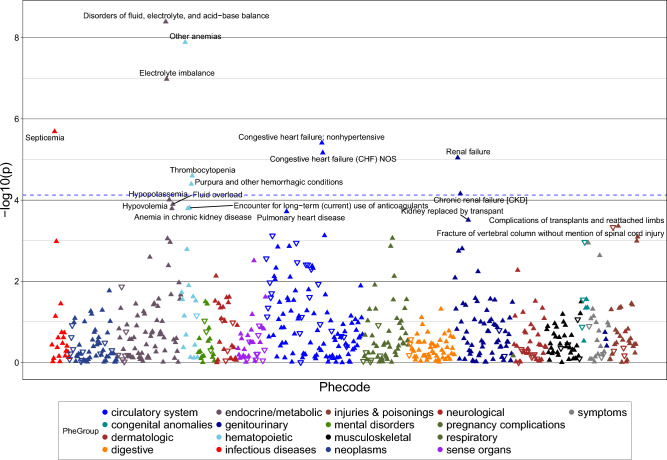
Phenome-Wide Association Study of the statistical interaction between SHAD and LEAN (*SHAD × LEAN*). LEAN is a binary variable indicating that a patient has 18.5 kg/m^2^ ≤ BMI < 25 kg/m^2^. Each coded phenotype is shown as a triangle and further grouped by color as indicated in the legend at the bottom. Upward pointing triangles indicate a positive association with SHAD*LEAN, while downward pointing triangles indicate a negative association. The blue dashed horizontal line on the graph indicates the level of statistical significance with Bonferroni multiple-comparison correction (*p* < 7.5 × 10^–5^). Phenotypes above the line are those that show a statistically significant association with SHAD*LEAN. The ten statistically significant phenotypes in order of statistical significance are the following: disorders of fluid, electrolyte, and acid–base balance; other anemias; electrolyte imbalance; septicemia; non-hypertensive congestive heart failure; congestive heart failure, not otherwise specified (NOS); renal failure; thrombocytopenia; purpura and other hemorrhagic conditions; chronic renal failure (CKD). *BMI* body mass index, *SHAD* spleen-hepatic attenuation difference.

A PheWAS plot with all 665 phenotypes is shown in Fig. 4, and Supplementary Table S13 lists the top 200 phenotypes in order of statistical significance. The PheWAS yielded a total of ten phecodes that met the Bonferroni-corrected threshold of *p* < 7.52 × 10^–5^ for statistical significance. The phenotype most strongly associated with *SHAD x LEAN* was disorders of fluid, electrolyte, and acid–base imbalance (*p* = 4.06 × 10^–9^), followed by anemia (*p* = 1.30 × 10^–8^). Circulatory and renal conditions were also significantly associated with *LEAN x SHAD*, with non-hypertensive congestive heart failure (*p* = 3.90 × 10^–6^) and unspecified congestive heart failure (*p* = 6.83 × 10^–6^) followed by renal failure (*p* = 9.02 × 10^–6^) and chronic kidney disease (CKD) (*p* = 6.96 × 10^–5^). Other significant associations included septicemia (*p* = 2.05 × 10^–6^), thrombocytopenia (*p* = 2.52 × 10^–5^), and purpura and other hemorrhagic conditions (*p* = 4.01 × 10^–5^).

A second PheWAS analysis was also conducted using SHAD and BMI as a continuous factor (*SHAD x BMI*) as shown in Supplementary Fig. S6. Gout and other gout related conditions were associated with *SHAD x BMI* (*p* < 2.4 × 10^–5^). Renal conditions became more significant, adding acute renal failure and kidney transplant, but the cardiovascular conditions fell just below the adjusted threshold for statistical significance.

### Validation of CT steatosis detection

Of the 8914 patients in our cohort, 400 patients had an MRI scan performed within 365 days of their CT scan. The correlation between the spleen-hepatic attenuation difference (SHAD) from CT and fat percentage from MRI resulted in the linear regression formula $SHAD\left(HU\right)=0.524\times Fat \, Percentage\left(\%\right)-9.101$, Pearson’s correlation coefficient r = 0.53, and *p* < 0.0001. The correlation between liver mean attenuation (LMA) from CT and fat percentage resulted in the linear regression formula $LMA\left(HU\right)= -0.679\times Fat \, Percentage\left(\%\right)+53.682$, r =  − 0.57, and *p* < 0.0001. In comparing CT-detected steatosis (SHAD ≥  − 1 HU or LMA < 40 HU) and MRI-detected steatosis (fat percentage > 5%), Cohen’s kappa coefficient was calculated to be 0.92. With only 400 patients from the cohort, there was insufficient statistical power to repeat the analyses from CT-detected steatosis, but there was strong agreement between CT- and MRI-detected steatosis for patients in our cohort with data from both modalities.

We repeated the PheWAS analysis for a cohort of 25,681 participants in the UK Biobank with both phecode data and proton density fat fraction (PDFF) calculations from MRI. The UK Biobank is largely composed of healthy participants, and thus the number of positive cases for relevant disease-related phecodes was significantly lower than in the PMBB cohort. Results are listed in Supplementary Table S14.

## Discussion

In this study, we sought to characterize the prevalence of lean hepatic steatosis and identify its associated conditions in a large health system biobank. We found associations between lean steatosis and cardiovascular conditions, metabolic diseases, and kidney disease and related complications. Additionally, our biomarker analysis showed that liver function in lean patients with steatosis is not significantly different from that of overweight patients with steatosis, besides increased ALP for lean patients. Though we were unable to measure fibrosis directly, we also observed a significant increase in the rate of progression of hepatic steatosis to fibrosis for lean patients as compared to overweight patients using the FIB-4 index.

We found that the prevalence of hepatic steatosis in our dataset was near that of other studies. In our dataset, we had a prevalence of 24.0% for hepatic steatosis, close to the estimated global rate of 25–30%^2,20^ and the rate from the CARDIA study with American patients^21^. 13.0% of patients with steatosis in our cohort had a lean BMI, lower than some other estimates of 16.7% and 25.3%^5,6^. Out of all lean patients, 13.0% had steatosis, slightly lower than estimates among nonobese patients from the Dionysos study, which was 16%^22^ and slightly higher than others^6^. Differences in our results may come from our patient population and the method we used to detect steatosis, as some of the other studies used different ways of measuring hepatic steatosis on CT or other imaging techniques such as MRI and US.

Across patients with steatosis, there is no strong consensus in the literature over the distribution between males and females. One study found that lean patients with steatosis are more likely to be female^4^, while another found that out of lean and overweight patients combined, men were significantly more likely to have NAFLD^23^. In our cohort there was no significant increase in the proportion of females in the lean steatosis group compared to the cohort overall. However, we did find that within both BMI categories, patients with steatosis in our cohort were significantly more likely to be male. Studies have also found differences in hepatic steatosis across racial groups. In one study, Hispanic patients were found to have the highest prevalence of NAFLD, while Black patients were found to have the lowest^23^. Another study found that Asian patients were more likely to develop hepatic steatosis, along with other metabolic diseases like T2DM, at lower BMIs compared to other racial groups^11^. Our cohort did not have sufficient representation from minority racial groups to conduct a comprehensive analysis of differences in hepatic steatosis across different racial groups, but among the overweight patients in our cohort, we found that Black patients had a significantly lower prevalence of hepatic steatosis than White patients while the opposite was true among lean patients.

As expected, based on the findings of previous studies^5^, a greater proportion of patients with steatosis were homozygous carriers of the PNPLA3 rs738409 variant compared to patients without steatosis. In our cohort, increased rates of genetic predisposition accompanied the increased severity of adverse metabolic factors in both lean and overweight patients with steatosis.

Concerning metabolic profile, we found that lean patients with steatosis had significantly higher rates of HTN, T2D, and HLD, and significantly lower average HDL than lean patients without steatosis, indicating that hepatic steatosis itself, independent from high BMI, is a strongly associated with metabolic disease risk. The association between metabolic syndrome and hepatic steatosis has been observed previously in an Austrian population^24^, and our study shows that the association also applies to a more racially heterogeneous American cohort. The association between lean steatosis and T2DM has previously been found to be particularly strong among Asian populations^11^, but the cohort in our study did not contain sufficient representation of Asian patients to test for this association. The association between hepatic steatosis and metabolic disease in our study was also reflected between overweight patients with steatosis compared to overweight patients without steatosis, showing that the increased risk for metabolic disease from hepatic steatosis holds across BMI categories. The one exception to this trend was that LDL values were observed to be the lower among lean patients with steatosis compared to lean patients without steatosis. It is possible that higher statin usage accounted for this observation in part, as lean patients with steatosis were more likely to have been prescribed a statin at some time before their scan (20.1 vs 16.6%). However, the power of this observation is limited in that the PMBB only notes that the medication was ordered and not whether the patient used the medication as prescribed or whether the patient stopped taking the medication for any reason.

Interestingly, metabolic profile was similar between lean patients with steatosis and overweight patients without steatosis. The similar metabolic laboratory values between lean patients with steatosis and overweight patients without steatosis may suggest that the presence of hepatic steatosis has a comparable contribution to determining the metabolic profile as being overweight alone.

We found that hepatic steatosis is associated with cardiovascular conditions independent from high BMI, confirming previous findings^25,26^. Congestive heart failure was independently associated with higher levels of hepatic fat in lean patients in the PheWAS, and lean patients with steatosis had a significantly higher rate of CVD than lean patients without steatosis. The increased risk of CVD associated with hepatic steatosis was even stronger for lean patients than overweight patients, suggesting the need to recognize lean steatosis as a CVD risk factor as well.

Previous studies have shown that CKD is also associated with NAFLD, even after controlling for several factors including BMI^7,9^. In our cohort, we found renal failure and CKD to be significantly associated with increased hepatic fat for lean patients in the PheWAS. Relatedly, anemia and electrolyte and acid–base imbalance were significantly associated with hepatic steatosis in lean patients. Anemia is known to be associated with CKD, especially in its later stages^27^, and loss of renal function can cause electrolyte and acid–base imbalance^28^. CKD also has a hand in altering metabolic processes, putting patients at greater risk of malnutrition and sarcopenia, with both dialysis and non-dialysis patients observed to have lower nutrient intakes^29^; this may be linked to its association with lean steatosis specifically. Renal failure is also known to be associated with cirrhosis of the liver^30^, but we did exclude for end stage liver disease in our dataset, which may indicate a risk for renal failure even in the less severe stages of liver disease. The exact mechanisms of the association between these renal conditions and hepatic steatosis remain unclear.

Alkaline phosphatase (ALP) was elevated in lean patients with steatosis, which was not seen in overweight patients with steatosis. Although we excluded common hepatobiliary conditions (aside from NAFLD) such as ESLD/cirrhosis and viral hepatitis, it is possible that lean patients with steatosis are more prone to have other hepatobiliary diseases, such as cholestasis or cholangitis. Notably, ALP can also be associated with non-hepatobiliary diseases. For instance, patients with chronic kidney disease typically have elevated alkaline phosphatase^31^. Of interest, several kidney-related diseases did cross the significance threshold in our PheWAS. However, further research is needed to investigate this finding.

Future studies should consider validating our results in another dataset to achieve greater generalizability. Using AI and non-invasive imaging would make it feasible to apply these techniques to a large cohort of patients. Although the number of individuals with lean steatosis in this study compared favorably with others in the literature^21,22^, it is acknowledged that there were comparatively fewer patients with lean steatosis compared to other groups such as overweight patients with or without steatosis. A potential future study combining data from multiple institutions may have even better statistical power for finding differences between groups.

Additionally, dietary factors and levels of physical activity are believed to impact hepatic steatosis risk^32^, and future studies should consider examining patient nutrition information in their analysis of hepatic steatosis to see if any associations can be made between hepatic fat, BMI, and nutrition. We are currently in the early stages of establishing a nutrition survey for a subset of our patients. Integrating image-derived abdominal adiposity measures using AI at scale may also yield results that improve our understanding of the nature of hepatic steatosis^33^, as increased visceral adiposity is known to be a risk factor for NAFLD^34^.

One limitation of this study was the dependence on administrative data such as ICD-9 codes to establish our associations. ICD-9 codes alone are an imperfect summary of patients’ conditions, especially for the purpose of applying exclusion criteria. Particularly, heavy drinking is known to significantly increase one’s risk for hepatic steatosis^35^, but the sensitivity of administrative data for alcohol dependence and alcohol abuse has been found to be low^36^. Additionally, this is a retrospective study analyzing only patients for whom medical tests were ordered, creating the possibility of a sample bias toward people with more health problems. The study only contained patients enrolled in the PMBB, which covers a single albeit large hospital system, and it was thus subject to the demographic constraints of the region.

In identifying patients with hepatic steatosis, though we used splenic and hepatic attenuation to quantify hepatic steatosis, CT is an imperfect way to measure it. CT has been found to have lower sensitivity than MRI for mild steatosis, and lower sensitivity than both MRI and US for moderate to severe steatosis^32^. However, the specificity of CT is higher than US and MRI for moderate to severe steatosis^35^, making it less likely to detect false positives. CT is also a non-invasive test and, like other imaging techniques such as MRI and US, is much more accessible than the alternative of liver biopsy to measure hepatic steatosis, which is infrequently performed for this indication in the United States. Even among the non-invasive imaging tests, CT is often the most widely accessible, which provided a large volume of cases for us to analyze retrospectively^12^. Among the 400 patients in our cohort with MRI scans within 365 days of CT, we found that SHAD and LMA are moderately correlated with fat percentage as measured on MRI (r = 0.53 for SHAD, r = -0.57 for LMA). We limited the date difference between the MRI and CT scans compared to 365 days, since it has been shown that the correlation between CT liver attenuation and MRI fat fraction worsens the longer the date difference^37^. As there were only 400 patients with data from both modalities available, there was insufficient statistical power to directly replicate our results with MRI in our cohort. However, when comparing steatosis detection between CT and MRI, the value of Cohen’s kappa coefficient (0.92) indicated very strong agreement between the two detection methods, providing confidence in our results from CT-based detection.

In our attempt to replicate our findings in a patient cohort with PDFF calculations available from the UK Biobank, we discovered that the prevalence of relevant disease-related phecodes was significantly lower than in the PMBB, as the UK Biobank contains a large proportion of healthy participants. The low prevalence of disease in the UK Biobank caused many of the associations involving the interaction between steatosis and the lean characteristic to lose significance, and we were thus unable to replicate the same results. This illustrates the advantages of the PMBB dataset in which the prevalence of various diseases is significantly higher as compared to the UK Biobank.

Many patients with steatosis are unaware that they have hepatic steatosis or NAFLD; the CARDIA study found that only 2.4% of patients with NAFLD were able to report a prior diagnosis, with the number being even lower among minorities^23^. AI applied to CT and MRI has the potential to raise hepatic steatosis awareness in patients, including whether they are at a higher risk for downstream metabolic consequences, by opportunistically screening them. This can help find lean patients with steatosis to ensure their representation in clinical trials. Overall, this study leveraged a deep learning-based analysis on a large academic biobank to further elucidate the phenotype of lean patients with steatosis, showing how this cohort differed from others in terms of associated blood biomarkers and clinical conditions, serving as a gateway for future translational discovery.

## Materials and methods

### Penn Medicine BioBank

Data from participants in the Penn Medicine BioBank (PMBB) were used to conduct this study. The PMBB integrates data from the electronic health record with imaging data and laboratory test results from enrolled patients at Penn Medicine, a hospital system based in Philadelphia, Pennsylvania. All PMBB participants gave informed consent for their data and test results to be used for future studies upon enrollment in the biobank. During the time that the data in this study was being collected, patients were enrolled through in-person encounters at outpatient Penn Medicine sites during which they provided written consent. All patients of at least 18 years of age who were able to give informed consent were eligible to enroll. The PMBB was approved by the Institutional Review Board at the University of Pennsylvania under IRB protocol 813,913. All research was conducted in accordance with relevant guidelines and regulations.

### Segmentation and liver fat quantification

**Figure 5:**
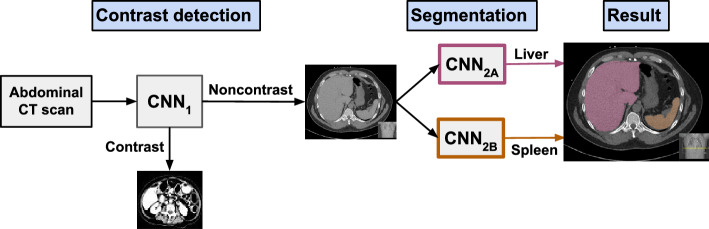
Deep learning pathway for hepatic steatosis quantification. CNN_1_ distinguishes between scans with and without intravenous contrast. CNN_2_ serves for organ segmentation, with CNN_2A_ segmenting the liver and CNN_2B_ segmenting the spleen. The resulting image demonstrates a representative segmentation of the liver (red) and spleen (orange) in the axial plane. The presence of hepatic steatosis is then quantified by the spleen hepatic attenuation difference (SHAD). SHAD is defined as Spleen Attenuation (HU) − Liver Attenuation (HU). *CNN* convolutional neural network, *HU* Hounsfield units.

Liver fat was quantified using a previously described fully automated deep learning approach^33^. In brief, for each complete CT study, the algorithm first determines from imaging metadata if the scan’s reconstructed images were provided in the axial (transverse) orientation. Images were further analyzed if the axial slice thickness was greater than 2 mm. Additionally, some series were excluded based on the DICOM field (00181210) “Convolution Kernel” as described elsewhere^38^. Unenhanced CT series were identified from pixel-level data using deep learning classification methods. Using only unenhanced CT scans was important in order to maintain consistency in comparing attenuation, since different tissues reach peak enhancement at different times in the presence of intravenous contrast. Liver and spleen segmentations were subsequently performed to determine absolute liver and spleen attenuation in HU and to determine the spleen-hepatic attenuation difference (SHAD), defined as the spleen attenuation minus the hepatic attenuation, to use as a measure of hepatic steatosis. A higher value for SHAD corresponds to increased hepatic steatosis. Outlier cases for which the liver mean attenuation (LMA) or the spleen mean attenuation were less than 0 HU or greater than 100 HU were excluded, as were those with SHAD greater than 30 HU or less than  − 30 HU. A summary of the deep learning pathway used is shown in Fig. 5. We used SHAD ≥  − 1 HU or LMA < 40 HU to identify patients with mild steatosis or greater^14,15^, and we repeated all analyses for patients with at least moderate-to-severe steatosis as defined by SHAD ≥ 10 HU or LMA < 40 HU.

### Cohort selection

Of the patients enrolled in the PMBB, 14,254 had abdominal CT scans from which it was possible to obtain a valid measurement of SHAD. If a patient had multiple scans, we chose the scan resulting in the highest SHAD measurement on which to base our analysis. Patients with missing age and race information, as well as those without a BMI measurement within 365 days of the study date of their selected CT scan, were excluded. We used phecodes, disease phenotypes mapped from ICD-9 codes, from the PheWAS catalog^39^ available online at https://phewascatalog.org/phecodes, to further exclude patients with diagnoses that may confound our analysis of BMI and NAFLD (Supplementary Table S15). Patients with alcohol use disorder and other diagnoses related to excessive alcohol consumption were excluded to remove patients potentially affected by alcoholic fatty liver disease, which was not the focus of this study. Patients with end-stage liver disease complications and viral hepatitis were excluded to remove the effects of those diseases on SHAD measurements. Patients with any cancer were excluded due to the risk of steatosis or steatohepatitis induced by certain chemotherapy drugs, as well as the high incidence of liver metastasis in common cancers such as colorectal cancer^40,41^. Patients with cachexia and those who had undergone bariatric surgery were excluded to minimize the effect of abnormal changes of BMI. The final cohort consisted of 8,914 patients. The number of cases remaining after each of the exclusion criteria is detailed in Supplementary Fig. S7. The cohort was dichotomized by BMI category (lean or overweight) and steatosis status determined by SHAD and liver mean attenuation (steatosis or no steatosis). Lean patients were those with 18.5 kg/m^2^ ≤ BMI < 25 kg/m^2^ while overweight patients had BMI ≥ 25 kg/m^2^.

### Validation of CT steatosis detection

All statistical analyses were performed using R (R Core Team, version 4.1.2; Foundation for Statistical Computing, Vienna, Austria). For patients in the cohort with available MRI scans performed within 365 days of their CT scan, the fat percentage was calculated based on the in-phase (IP) and out-of-phase (OP) signal intensity from the liver and spleen, as manually annotated by a board-certified radiologist with over 10 years of experience in radiology, according to Eq. (1):

1 $$Fat\,Percentage = 100 \times \frac{{\left( {LiverIP/SpleenIP} \right) - \left( {LiverOP/SpleenOP} \right)}}{{2\left( {LiverIP/SpleenIP} \right)}}$$

A linear regression was performed for SHAD versus fat percentage and LMA versus fat percentage. The Pearson product-moment correlation was performed to calculate the corresponding Pearson’s correlation coefficient and p-value for each comparison. On MRI, a fat percentage of > 5% is considered clinically significant steatosis. Using the thresholds of fat percentage > 5% for MRI-detected steatosis and SHAD ≥  − 1 HU or LMA < 40 HU for CT-detected steatosis, Cohen’s Kappa coefficient was calculated for the two methods of hepatic steatosis detection.

### Cohort analysis

Hepatic steatosis was defined as SHAD ≥ -1 HU or LMA < 40 HU. All analyses were repeated for the cutoff of moderate-to-severe hepatic steatosis, as defined by SHAD ≥ 10 HU or LMA < 40 HU and for the cohort split into three BMI categories, separating obese patients from overweight patients (lean = 18.5 kg/m^2^ ≤ BMI < 25 kg/m^2^, overweight = 25 kg/m^2^ ≤ BMI < 30 kg/m^2^, obese = BMI ≥ 30 kg/m^2^). Clinical and demographic statistics were determined for each cohort, including age, self-reported sex and race, BMI, liver mean attenuation (LMA), SHAD, cardiovascular disease: including ischemic heart disease and atherosclerosis (CVD), hypertension (HTN), type 2 diabetes mellitus (T2DM), and hyperlipidemia (HLD) as determined by ICD-9 codes. Differences in the demographic and clinical characteristics between the patient groups were determined by chi-square testing for categorical variables (sex, race, CVD, HTN, T2DM, HLD) and the Kruskal–Wallis test for continuous variables (age). BMI, LMA, and SHAD were not tested with the Kruskal–Wallis test because they were the factors that defined the criteria for dichotomization in the first place. *p*-values were adjusted with the Benjamini–Hochberg method to control for the false discovery rate, and *p* < 0.05 was considered to show statistical significance. All variables also underwent post-hoc pairwise chi-square testing to determine between which pairs of groups there existed a statistically significant difference. Additionally, the proportion of patients in each BMI category carrying the PNPLA3 rs738409 variant was calculated, with those whose genetic data was available, and *p*-values between the groups were calculated using the chi-squared test.

### Blood biomarker analysis

Clinical laboratory results for patients in the cohort were used to compare values between the 4 groups (lean with steatosis, lean without steatosis, overweight with steatosis, overweight without steatosis) for the eight different biomarkers related to metabolism and liver function: hemoglobin A1c (A1c), alkaline phosphatase (ALP), alanine transaminase (ALT), aspartate transaminase (AST), total cholesterol (Cholesterol), high-density lipoprotein cholesterol (HDL), low-density lipoprotein cholesterol (LDL), and triglycerides (TG). For each patient, laboratory results from the date closest to their CT scan date were used for the analysis. Laboratory results acquired greater than 90 days before or after the scan were excluded. P-values were determined using the Kruskal–Wallis test between the groups. The Benjamini–Hochberg method was used to adjust the p-values to control for the false discovery rate, and *p* < 0.05 was considered to show statistical significance. Dunn’s test for multiple comparisons was conducted post-hoc for each of the biomarkers.

In order to assess the progression of hepatic steatosis to fibrosis, the FIB-4 index was calculated according to Eq. (2):

2 $$FIB - 4 = Age(years) \times AST(U/L)/[PLT(10^{9} /L) \times \sqrt {ALT} (U/L)]$$

A FIB-4 score of ≥ 3.25 has been proposed as a conservative threshold to indicate severe fibrosis^19^. The distribution of FIB-4 scores was compared between lean patients with steatosis and overweight patients with steatosis using the two sample Wilcoxon test.

All analyses were repeated for the cutoff of moderate-to-severe hepatic steatosis, as defined by SHAD ≥ 10 HU or LMA < 40 HU and for the cohort split into three BMI categories, separating obese patients from overweight patients (lean = 18.5 kg/m^2^ ≤ BMI < 25 kg/m^2^, overweight = 25 kg/m^2^ ≤ BMI < 30 kg/m^2^, obese = BMI ≥ 30 kg/m^2^).

### Phenome-wide association study

A phenome-wide association study (PheWAS)^42^ was conducted over the patient cohort using the PheWAS R package to determine the relationship between each phecode and the statistical interaction between the binary characteristic of being lean (LEAN) and the continuous value of SHAD. Patients having a BMI of at least 18.5 kg/m^2^ but less than 25 kg/m^2^ were defined as lean. There were 1816 phecodes in total used across the cohort. Only phecodes with at least 100 instances among the patient cohort were included, leaving 665 phecodes to be used in the PheWAS. For each phecode, a logistic regression model was fitted to the interaction of hepatic fat values and the lean characteristic of the patient cohort and their main effects, controlling for age, sex, and race according to Eq. (3):

3 $$log\left( {\frac{{p\left( {PHE = 1} \right)}}{{p\left( {PHE = 0} \right)}}} \right) = SHAD \times LEAN + SHAD + LEAN + AGE + SEX + RACE$$

where *p*(*PHE* = 1) is the probability of a patient expressing the phenotype, and *p*(*PHE* = 0) is the probability of a patient not expressing the phenotype. The statistical interaction between the lean characteristic and SHAD (*SHAD x LEAN*) was used to determine the relationship between each phecode and hepatic fat values for lean patients specifically, isolating it from the effects of other values that varied within the cohort. The Bonferroni correction was used to determine a corrected significance threshold of p < 7.52 × 10^–5^ to reduce the possibility of type I errors, the typical correction for a PheWAS analysis. The PheWAS was repeated using the same procedure as described above for the statistical interaction between SHAD and the continuous variable BMI according to Eq. (4):

4 $$log\left( {\frac{{p\left( {PHE = 1} \right)}}{{p\left( {PHE = 0} \right)}}} \right) = SHAD \times BMI + SHAD + BMI + AGE + SEX + RACE$$

### Supplementary Information

Supplementary Information.

## Data Availability

As clinical data from a biobank was used for this study, it is not possible to make the data available publicly. However, deidentified data could be shared with a qualified researcher upon request, pursuant to the rules and regulations of the biobank and the existing IRB. Please contact Hersh Sagreiya to request data from the study. Models will be made available online.
