# Supplementary material for: Clinical correlates of CT imaging-derived phenotypes among lean and overweight patients with hepatic steatosis
Source: Sci Rep. 2024 Jan 2;14:53. doi: 10.1038/s41598-023-49470-x (PMC10761858; doi:10.1038/s41598-023-49470-x)
Supplement: Supplementary file 1 — Supplementary Information. [file 41598_2023_49470_MOESM1_ESM.pdf]

Clinical correlates of CT imaging-derived phenotypes among lean and overweight patients with hepatic steatosis

Isabel Song[1], Elizabeth W. Thompson[1], Anurag Verma[2], Matthew T. MacLean[1,3], Jeffrey Duda[1], Ameena Elahi[1], Richard Tran[1], Pavan Raghupathy[1], Sophia Swago[1], Mohamad Hazim[1], Abhijit Bhattaru[1], Carolin Schneider[2], Marijana Vujkovic[3], Drew A. Torigian[1], Charles E. Kahn[1], James C. Gee[1], Arijitt Borthakur[1], Colleen M. Kripke[2], Christopher C. Carson[2], Rotonya Carr[2,4], Qasim Jehangir[2], Yi-An Ko[3], Harold Litt[1], Mark Rosen[1], David A. Mankoff[1], Mitchell D. Schnall[1], Haochang Shou[5], Julio Chirinos[2], Scott M. Damrauer[6], Marina Serper[2], Jinbo Chen[5], Daniel J. Rader[7], Penn Medicine BioBank, Walter R. T. Witschey[1], Hersh Sagreiya[1]*

[1]Department of Radiology, Perelman School of Medicine, University of Pennsylvania, Philadelphia, PA, USA
[2]Department of Medicine, Perelman School of Medicine, University of Pennsylvania, Philadelphia, PA, USA
[3]Department of Genetics, Perelman School of Medicine, University of Pennsylvania, Philadelphia, PA, USA
[4]Department of Medicine, School of Medicine, University of Washington, Seattle, WA, USA
[5]Department of Biostatistics, Epidemiology, and Informatics, Perelman School of Medicine, University of Pennsylvania, Philadelphia, PA, USA
[6]Department of Surgery, Perelman School of Medicine, University of Pennsylvania, Philadelphia, PA, USA
[7]Institute for Translational Medicine and Therapeutics, Perelman School of Medicine, University of Pennsylvania, Philadelphia, PA, USA

Address correspondence to:
Hersh Sagreiya
Hospital of the University of Pennsylvania
Department of Radiology,
Division of Abdominal Imaging
3400 Spruce Street
Philadelphia, PA 19104
Email: hersh.sagreiya@pennmedicine.upenn.edu

Supplementary Table S1: Uncorrected p-values from pairwise chi-square testing for clinical and demographic categorical characteristics for mild to severe steatosis.

	Lean with steatosis vs Lean without steatosis	Lean with steatosis vs Overweight with steatosis	Lean without steatosis vs Overweight with steatosis	Lean with steatosis vs Overweight without steatosis	Lean without steatosis vs Overweight without steatosis	Overweight with steatosis vs overweight without steatosis
	P-value	P-value	P-value	P-value	P-value	P-value
**Sex**	0.014	6.6E-3	<0.0001	0.52	<0.0001	<0.0001
**Race**	0.019	0.020	<0.0001	5.5E-3	<0.0001	3.4E-4
**CVD**	<0.0001	0.15	<0.0001	6.5E-3	<0.0001	8.1E-3
**HTN**	<0.0001	0.026	<0.0001	0.32	<0.0001	<0.0001
**T2DM**	<0.0001	<0.0001	<0.0001	0.78	<0.0001	<0.0001
**HLD**	1.3E-3	6.4E-3	<0.0001	0.29	<0.0001	1.3E-4

Lean patients have 18.5 $kg/m^2 \leq BMI < 25$ kg/m^2. Patients with steatosis have SHAD≥10 HU or LMA<40 HU. BMI=body mass index, SHAD=spleen-hepatic attenuation difference, LMA=liver mean attenuation, CVD=cardiovascular disease, HTN=hypertension, T2DM=type 2 diabetes mellitus, HLD=hyperlipidemia

Supplementary Table S2: Demographic and clinical characteristics of the patient cohort used in the study, considering only moderate-to-severe steatosis.

		Lean ($18.5\ kg/m^2 \leq$ BMI < $25\ kg/m^2$)		Overweight (BMI $\geq 25\ kg/m^2$)		
	Total (N=8914)	With steatosis (N=120)	Without steatosis (N=2025)	With steatosis (N=1237)	Without steatosis (N=5532)	P-value
Age (years)	63 [19]	66 [19]	62 [22]	62 [15]	63 [18]	0.016
Sex (n (%))						
Male	4895 (54.9%)	69 (57.3%)	929 (45.9%)	780 (63.1%)	3117 (56.3%)	<0.0001
Female	4019 (45.1%)	51 (42.5%)	1096 (54.1%)	457 (36.9%)	2415 (43.7%)	
Race (n (%))						
White	6396 (71.8%)	89 (74.2%)	1550 (76.5%)	933 (75.4%)	3824 (69.1%)	
Black	2031 (22.8%)	20 (16.7%)	336 (16.6%)	242 (19.6%)	1433 (25.9%)	<0.0001
Asian	144 (1.6%)	3 (2.5%)	56 (2.8%)	16 (1.3%)	69 (1.2%)	
Other/Unknown	343 (3.8%)	8 (6.7%)	83 (4.1%)	46 (3.7%)	206 (3.7%)	
BMI (kg/m^2)	28 [8]	23 [3]	22 [2]	33 [9]	29 [6]	-
LMA (HU)	51.50 [12.30]	35.89 [8.09]	55.33 [9.10]	34.59 [9.22]	52.43 [9.86]	-
SHAD (HU)	-8.09 [10.32]	8.31 [11.67]	-9.68 [8.05]	4.94 [14.42]	-9.31 [9.06]	-
CVD (n (%))	3001 (33.7%)	48 (40.0%)	587 (29.0%)	459 (37.1%)	1907 (34.5%)	<0.0001
HTN (n (%))	5676 (63.7%)	83 (69.2%)	1038 (51.3%)	914 (73.9%)	3641 (65.8%)	<0.0001
T2DM (n (%))	2482 (27.8%)	35 (29.2%)	339 (16.7%)	553 (44.7%)	1555 (28.1%)	<0.0001
HLD (n (%))	5120 (57.4%)	75 (62.5%)	956 (47.2%)	776 (62.7%)	3313 (59.9%)	<0.0001

Age, BMI, LMA, and SHAD values listed for each group are in the format of median [interquartile range]. A higher value for SHAD corresponds to increased hepatic steatosis. P-values were determined using the Kruskal-Wallis test for continuous fields (age) and chi-square for categorical fields (sex, race, CVD, HTN, T2DM, HLD) and were corrected using the Benjamini-Hochberg method for a significance threshold of $p<0.05$. P-values were not calculated for BMI, LMA, and SHAD because the groups were dichotomized by these quantities. Lean patients have $18.5\ kg/m^2 \leq BMI < 25\ kg/m^2$. Patients with steatosis have SHAD≥10 HU or LMA<40 HU. BMI=body mass index, LMA=liver mean attenuation, SHAD=spleen-hepatic attenuation difference, CVD=cardiovascular disease, HTN=hypertension, T2DM=type 2 diabetes mellitus, HLD=hyperlipidemia.

Supplementary Table S3: Chi-squared and p-values from pairwise chi-square testing for clinical and demographic categorical characteristics, considering only moderate-to-severe steatosis.

	Lean with steatosis vs Lean without steatosis		Lean with steatosis vs Overweight with steatosis		Lean without steatosis vs Overweight with steatosis		Lean with steatosis vs Overweight without steatosis		Lean without steatosis vs Overweight without steatosis		Overweight with steatosis vs Overweight without steatosis	
	X^2	P-value	X^2	P-value	X^2	P-value	X^2	P-value	X^2	P-value	X^2	P-value
**Sex**	5.69	**0.029**	1.21	0.35	90.17	**<0.0001**	0.03	0.92	64.89	**<0.0001**	18.37	**<0.0001**
**Race**	1.87	0.70	4.02	0.35	11.77	**0.015**	8.46	0.058	87.57	**<0.0001**	22.15	**1.5E-4**
**CVD**	5.07	**0.040**	0.12	0.83	22.12	**<0.0001**	0.89	0.43	19.84	**<0.0001**	2.69	0.14
**HTN**	14.18	**3.5E-4**	2.81	0.14	203.22	**<0.0001**	0.07	0.86	180.71	**<0.0001**	35.47	**<0.0001**
**T2DM**	10.45	**2.5E-3**	14.41	**3.3E-4**	330.5	**<0.0001**	0.00	1.0	117.1	**<0.0001**	136.59	**<0.0001**
**HLD**	8.32	**7.4E-3**	0.39	0.64	89.11	**<0.0001**	0.00	1.0	123.28	**<0.0001**	3.61	0.086

P-values were adjusted with the Benjamini-Hochberg method for a $p<0.05$ level of statistical significance. Lean patients have 18.5 kg/m^2≤BMI<25 kg/m^2. Patients with steatosis have SHAD≥10 HU or LMA<40 HU. BMI=body mass index, SHAD=spleen-hepatic attenuation difference, LMA=liver mean attenuation, CVD=cardiovascular disease, HTN=hypertension, T2DM=type 2 diabetes mellitus, HLD=hyperlipidemia.

Supplementary Table S4: Carriers of the PNPLA3 I148M variant accounting for moderate-to-severe steatosis.

	Lean with steatosis (N=84)	Lean without steatosis (N=1494)	Overweight with steatosis (N=938)	Overweight without steatosis (N=4250)
Non-carrier	46 (54.8%)	933 (62.4%)	497 (53.0%)	2743 (64.5%)
Heterozygous	29 (34.5%)	486 (32.5%)	354 (37.7%)	1347 (31.7%)
Homozygous	9 (10.7%)	75 (5.0%)	87 (9.3%)	160 (3.8%)

Lean patients have 18.5 $kg/m^2 \leq BMI < 25$ kg/m^2. Patients with steatosis have SHAD≥10 HU or LMA<40 HU. The rate of patients who are homozygous carriers by the chi-squared test: $p=0.044$ for lean patients with vs without steatosis, $p<0.0001$ for overweight patients with vs without steatosis, and 0.81 for lean vs overweight patients with steatosis.

Supplementary Table S5: Demographic and clinical characteristics of the patient cohort used in the study, split into three BMI categories.

		Lean ($18.5 \leq$ BMI < 25 kg/m^2)		Overweight ($25 \leq$ BMI < 30 kg/m^2)		Obese (BMI $\geq$ 30 kg/m^2)	
	Total (N=8914)	With steatosis (N=120)	Without steatosis (N=2025)	With steatosis (N=575)	Without steatosis (N=2588)	With steatosis (N=1288)	Without steatosis (N=2318)
Age (years)	63 [19]	65.5 [20.75]	62 [21]	64 [14]	64 [18]	61 [16]	61 [18]
Sex (n (%))							
Male	4895 (54.9%)	149 (53.6%)	849 (45.5%)	382 (66.4%)	1602 (61.9%)	779 (60.5%)	1134 (48.9%)
Female	4019 (45.1%)	129 (46.4%)	1018 (54.5%)	193 (33.6%)	986 (38.1%)	509 (39.5%)	1184 (51.1%)
Race (n (%))							
White	6396 (71.8%)	193 (69.4%)	1446 (77.5%)	47 (77.7%)	1951 (75.4%)	931 (72.3%)	1428 (61.6%)
Black	2031 (22.8%)	59 (21.2%)	297 (15.9%)	94 (16.3%)	494 (19.1%)	300 (23.3%)	787 (34.0%)
Asian	144 (1.6%)	8 (2.9%)	51 (2.7%)	11 (1.9%)	46 (1.8%)	13 (1.0%)	15 (0.6%)
Other	343 (3.8%)	18 (6.5%)	73 (3.9%)	23 (4.0%)	97 (3.7%)	44 (3.4%)	88 (3.8%)
BMI (kg/m^2)	28 [8]	23 [3]	22 [2.5]	27 [2]	27 [2]	35 [7]	33 [6]
LMA (HU)	51.50 [12.3]	42.43 [10.19]	55.86 [8.63]	39.56 [10.31]	54.16 [8.82]	36.81 [11.43]	52.12 [9.65]
SHAD (HU)	-8.09 [10.32]	2.44 [7.15]	-10.16 [7.27]	2.58 [7.56]	-10.05 [7.54]	3.02 [10.23]	-10.28 [8.46]
CVD (n (%))	3001 (33.7%)	116 (41.7%)	519 (27.8%)	228 (39.7%)	887 (34.3%)	469 (36.4%)	782 (33.7%)
HTN (n (%))	5676 (63.7%)	191 (68.7%)	930 (49.8%)	405 (70.4%)	1582 (61.1%)	966 (75.0%)	1602 (69.1%)
T2DM (n (%))	2482 (27.8%)	81 (29.1%)	293 (15.7%)	204 (35.5%)	563 (21.8%)	589 (45.7%)	752 (32.4%)
HLD (n (%))	5120 (57.4%)	160 (57.6%)	871 (46.7%)	378 (65.7%)	1515 (58.5%)	809 (62.8%)	1387 (59.8%)

P<0.0001 for all relevant rows. Age, BMI, LMA, and SHAD values listed for each group are in the format of median [interquartile range]. A higher value for SHAD corresponds to increased hepatic steatosis. P-values were determined using the Kruskal-Wallis test for continuous fields (age) and chi-square for categorical fields (sex, race, CVD, HTN, T2DM, HLD) and were corrected using Benjamini-Hochberg method for a significance threshold of p<0.05. Lean patients have 18.5 kg/m^2≤BMI<25 kg/m^2, overweight patients have 25 kg/m^2≤BMI<30 kg/m^2, and obese patients have BMI≥30 kg/m^2. Patients with steatosis have SHAD≥-1 HU or LMA<40 HU. BMI=body mass index, LMA=liver mean attenuation, SHAD=spleen-hepatic attenuation difference, CVD=cardiovascular disease, HTN=hypertension, T2DM=type 2 diabetes mellitus, HLD=hyperlipidemia.

Supplementary Table S6: Chi-squared and p-values from pairwise chi-square testing for clinical and demographic categorical characteristics with the cohort split into three BMI categories.

A. Lean vs obese patients

	Lean with steatosis vs Obese with steatosis		Lean without steatosis vs Obese with steatosis		Lean with steatosis vs Obese without steatosis		Lean without steatosis vs Obese without steatosis	
	X^2	P-value	X^2	P-value	X^2	P-value	X^2	P-value
Sex	4.21	0.054	68.13	**<0.0001**	1.99	0.19	4.79	**0.040**
Race	12.04	**0.013**	36.31	**<0.0001**	33.07	**<0.0001**	196.32	**<0.0001**
CVD	2.98	0.11	26.40	**<0.0001**	7.74	**9.9E-3**	16.19	**1.3E-4**
HTN	7.84	**9.6E-3**	250.64	**<0.0001**	0.081	0.79	199.84	**<0.0001**
T2DM	35.39	**<0.0001**	374.60	**<0.0001**	2.74	0.12	173.93	**<0.0001**
HLD	5.75	**0.024**	100.64	**<0.0001**	1.05	0.35	80.56	**<0.0001**

B. Overweight vs obese patients

	Overweight with steatosis vs Obese with steatosis		Overweight without steatosis vs Obese with steatosis		Overweight with steatosis vs Obese without steatosis		Overweight without steatosis vs Obese without steatosis	
	X^2	P-value	X^2	P-value	X^2	P-value	X^2	P-value
Sex	5.75	**0.024**	0.67	0.45	55.95	**<0.0001**	82.99	**<0.0001**
Race	13.56	**7.1E-3**	12.13	**0.012**	73.65	**<0.0001**	149.75	**<0.0001**
CVD	1.78	0.22	1.48	0.26	7.63	**0.010**	0.34	0.58
HTN	6.18	**0.020**	82.89	**<0.0001**	0.44	0.54	32.78	**<0.0001**
T2DM	23.37	**<0.0001**	251.00	**<0.0001**	0.79	0.41	76.17	**<0.0001**
HLD	0.82	0.41	6.75	**0.016**	8.42	**7.3E-3**	1.78E-29	1.0

P-values were adjusted with the Benjamini-Hochberg method for a $p<0.05$ level of statistical significance. Lean patients have 18.5 $kg/m^2 \leq BMI < 25$ kg/m^2, overweight patients have 25 $kg/m^2 \leq BMI < 30$ kg/m^2, and obese patients have $BMI \geq 30$ kg/m^2. Patients with steatosis have SHAD≥-1 HU or LMA<40 HU. BMI=body mass index, SHAD=spleen-hepatic attenuation difference, LMA=liver mean attenuation, CVD=cardiovascular disease, HTN=hypertension, T2DM=type 2 diabetes mellitus, HLD=hyperlipidemia.

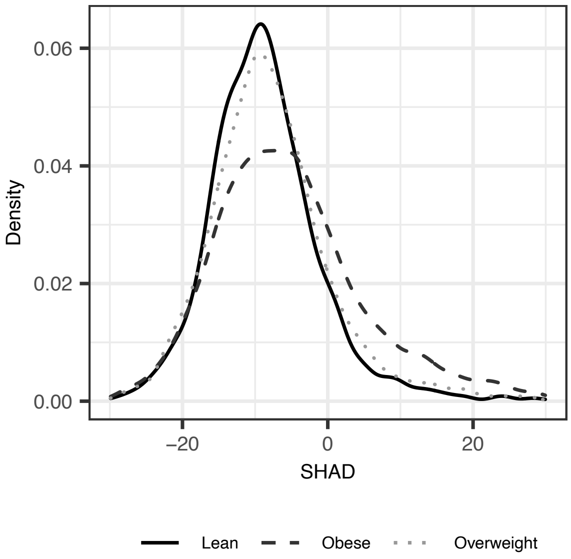

Supplementary Figure S1: Density of SHAD values for lean, overweight, and obese patients. P<0.0001 as determined by the Wilcoxon test. Lean patients have 18.5 kg/m^2≤BMI<25 kg/m^2, overweight patients have 25 kg/m^2≤BMI<30 kg/m^2, and obese patients have BMI≥30 kg/m^2. Patients with steatosis have SHAD≥-1 HU or LMA<40 HU. BMI=body mass index, SHAD=spleen-hepatic attenuation difference, LMA=liver mean attenuation.

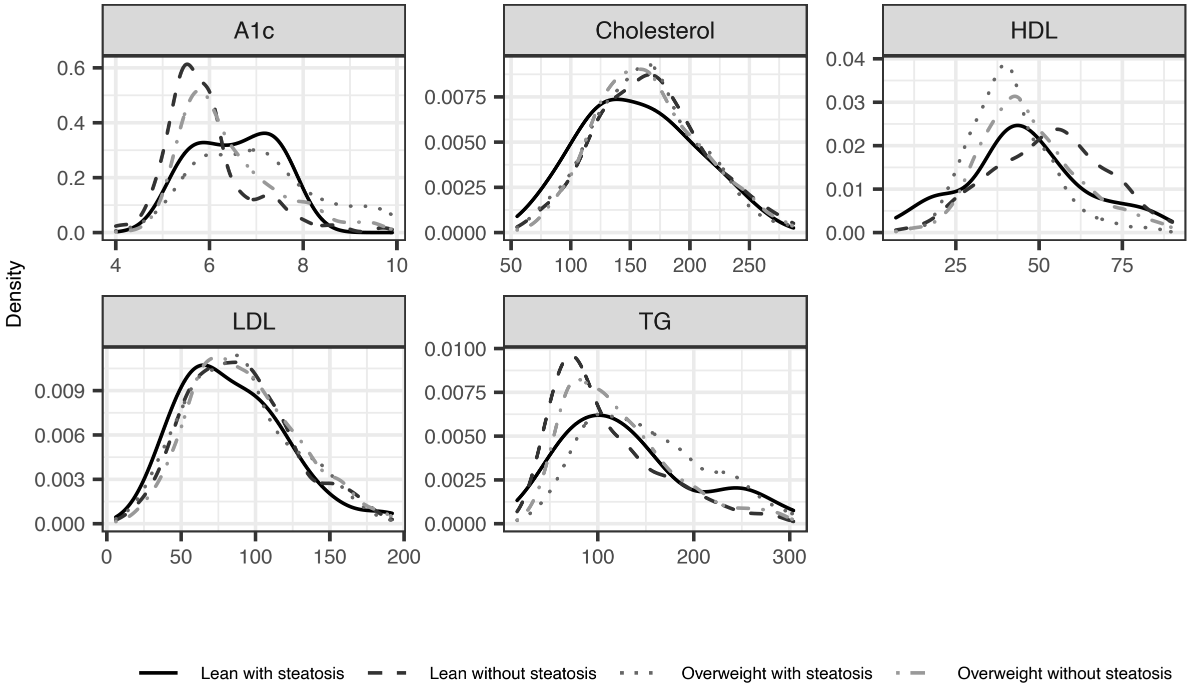

Supplementary Figure S2: Metabolic blood biomarker distributions of the patient cohorts, considering only moderate-to-severe steatosis. Lean patients have 18.5 kg/m^2≤BMI<25 kg/m^2. Patients with steatosis have SHAD≥10 HU or LMA<40 HU. BMI=body mass index, SHAD=spleen-hepatic attenuation difference, LMA=liver mean attenuation, A1c=hemoglobin A1c, HDL=high-density lipoprotein, LDL=low-density lipoprotein, TG=triglycerides.

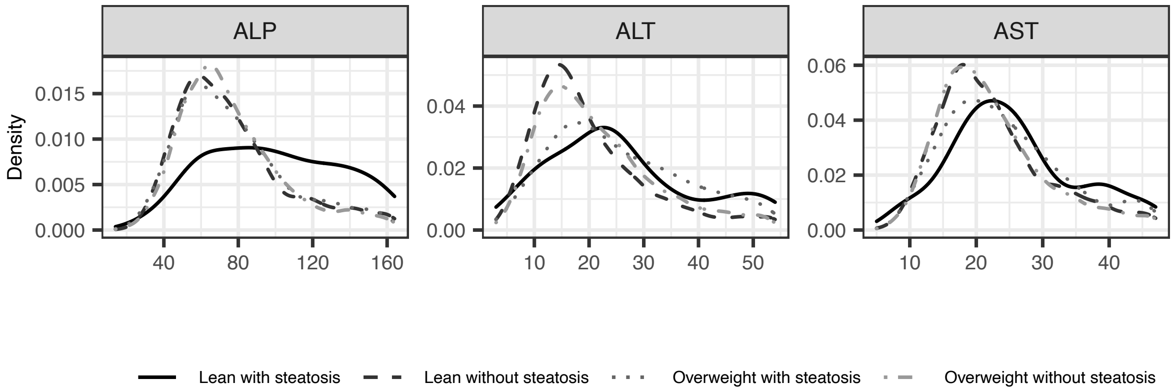

Supplementary Figure S3: Liver function biomarker distributions of the patient cohorts, considering only moderate-to-severe steatosis. Lean patients have 18.5 $kg/m^2 \leq BMI < 25$ kg/m^2. Patients with steatosis have SHAD≥10 HU or LMA<40 HU. BMI=body mass index, SHAD=spleen-hepatic attenuation difference, LMA=liver mean attenuation, ALP=alkaline phosphatase, ALT=alanine transaminase, AST=aspartate transaminase.

Supplementary Table S7: Uncorrected p-values from Dunn's test for multiple comparisons for biomarkers between the four groups of the patient cohort for mild to severe steatosis.

	Lean with steatosis vs Lean without steatosis	Lean with steatosis vs Overweight with steatosis	Lean without steatosis vs Overweight with steatosis	Lean with steatosis vs Overweight without steatosis	Lean without steatosis vs Overweight without steatosis	Overweight with steatosis vs overweight without steatosis
	P-value	P-value	P-value	P-value	P-value	P-value
A1c (%)	0.022	0.22	<0.0001	0.64	<0.0001	<0.0001
Cholesterol (mg/dL)	3.0E-3	0.056	0.043	0.011	0.22	0.22
HDL (mg/dL)	<0.0001	0.077	<0.0001	0.22	<0.0001	<0.0001
LDL (mg/dL)	8.6E-3	0.023	0.50	8.9E-4	0.19	0.034
TG (mg/dL)	1.0E-3	4.3E-3	<0.0001	0.41	<0.0001	<0.0001
ALP (U/L)	<0.0001	<0.0001	5.5E-3	<0.0001	0.095	0.10
ALT (U/L)	<0.0001	0.023	<0.0001	0.014	<0.0001	<0.0001
AST (U/L)	<0.0001	0.061	<0.0001	<0.0001	0.45	<0.0001

Lean patients have $18.5\ kg/m^2 \leq BMI < 25\ kg/m^2$. Patients with steatosis have SHAD≥-1 HU or LMA<40 HU. BMI=body mass index, SHAD=spleen-hepatic attenuation difference, LMA=liver mean attenuation, A1c=hemoglobin A1c, ALP=alkaline phosphatase, ALT=alanine transaminase, AST=aspartate transaminase, HDL=high-density lipoprotein, LDL=low-density lipoprotein, TG=triglycerides.

Supplementary Table S8: Biomarker mean values and number of samples over the patient cohort, considering only moderate-to-severe steatosis.

	Lean with steatosis		Lean without steatosis		Overweight with steatosis		Overweight without steatosis		
	N	Median [IQR]	N	Median [IQR]	N	Median [IQR]	N	Median [IQR]	P-value
A1c (%)	9	6.6 [1.6]	111	5.7 [0.8]	123	6.9 [1.7]	419	6.1 [1.3]	<0.0001
Cholesterol (mg/dL)	40	156 [66.25]	557	166 [62]	358	162.5 [57]	1626	162 [61]	0.47
HDL (mg/dL)	36	44.5 [20.5]	494	54 [23]	342	40 [14]	1524	45 [18]	<0.0001
LDL (mg/dL)	37	77 [46]	521	86 [49]	325	87 [48]	1519	89 [49]	0.045
TG (mg/dL)	42	114.5 [89.25]	624	91.5 [72]	396	141 [92]	1747	110 [74]	<0.0001
ALP (U/L)	85	96 [60]	1229	70 [35]	777	71 [37]	3068	70 [33]	<0.0001
ALT (U/L)	95	24 [17.5]	1389	17 [12]	827	23 [18]	3567	19 [13]	<0.0001
AST (U/L)	91	24 [11]	1364	21 [10]	827	23 [13]	3512	21 [10]	<0.0001

P-values were calculated using the Kruskal-Wallis test and corrected using Benjamini-Hochberg method for a threshold of p<0.05 for statistical significance. Lean patients have 18.5 $kg/m^2 \leq BMI < 25\ kg/m^2$. Patients with steatosis have SHAD≥10 HU or LMA<40 HU. BMI=body mass index, SHAD=spleen-hepatic attenuation difference, LMA=liver mean attenuation, IQR=interquartile range, A1c=hemoglobin A1c, ALP=alkaline phosphatase, ALT=alanine transaminase, AST=aspartate transaminase, HDL=high-density lipoprotein, LDL=low-density lipoprotein, TG=triglycerides.

Supplementary Table S9: Z-scores and p-values from Dunn's test for multiple comparisons for biomarkers between the four groups of the patient cohort by the Kruskal-Wallis test, considering only moderate-to-severe steatosis.

	Lean with steatosis vs Lean without steatosis		Lean with steatosis vs Overweight with steatosis		Lean without steatosis vs Overweight with steatosis		Lean with steatosis vs Overweight without steatosis		Lean without steatosis vs Overweight without steatosis		Overweight with steatosis vs Overweight without steatosis	
	Z	P	Z	P	Z	P	Z	P	Z	P	Z	P
A1c (%)	1.91	0.10	-0.83	0.50	-7.24	**<0.0001**	0.61	0.60	-4.26	**<0.0001**	4.81	**<0.0001**
Cholesterol (mg/dL)	-1.34	0.28	-0.98	0.47	1.05	0.41	-1.13	0.39	0.81	0.50	-0.54	0.64
HDL (mg/dL)	-2.56	**0.023**	1.89	0.10	10.97	**<0.0001**	-0.37	0.75	7.33	**<0.0001**	-6.56	**<0.0001**
LDL (mg/dL)	-0.91	0.47	-0.69	0.56	0.49	0.66	-1.50	0.22	-1.88	0.10	-2.14	0.063
TG (mg/dL)	2.45	**0.030**	-2.19	0.057	-11.59	**<0.0001**	0.73	0.54	-5.9	**<0.0001**	8.43	**<0.0001**
ALP (U/L)	6.19	**<0.0001**	5.72	**<0.0001**	-0.89	0.47	5.99	**<0.0001**	-1.06	0.41	0.12	0.90
ALT (U/L)	4.57	**<0.0001**	-0.14	0.90	-11.38	**<0.0001**	3.21	**3.0E-3**	-4.77	**<0.0001**	9.05	**<0.0001**
AST (U/L)	3.33	**2.1E-3**	1.42	0.25	-4.61	**<0.0001**	3.66	**6.4E-4**	0.89	0.47	5.99	**<0.0001**

P-values were corrected using the Benjamini-Hochberg method for a threshold of $p<0.05$ for statistical significance. Lean patients have 18.5 $kg/m^2 \leq BMI < 25\ kg/m^2$. Patients with steatosis have SHAD≥10 HU or LMA<40 HU. BMI=body mass index, SHAD=spleen-hepatic attenuation difference, LMA=liver mean attenuation, A1c=hemoglobin A1c, ALP=alkaline phosphatase, ALT=alanine transaminase, AST=aspartate transaminase, HDL=high-density lipoprotein, LDL=low-density lipoprotein, TG=triglycerides.

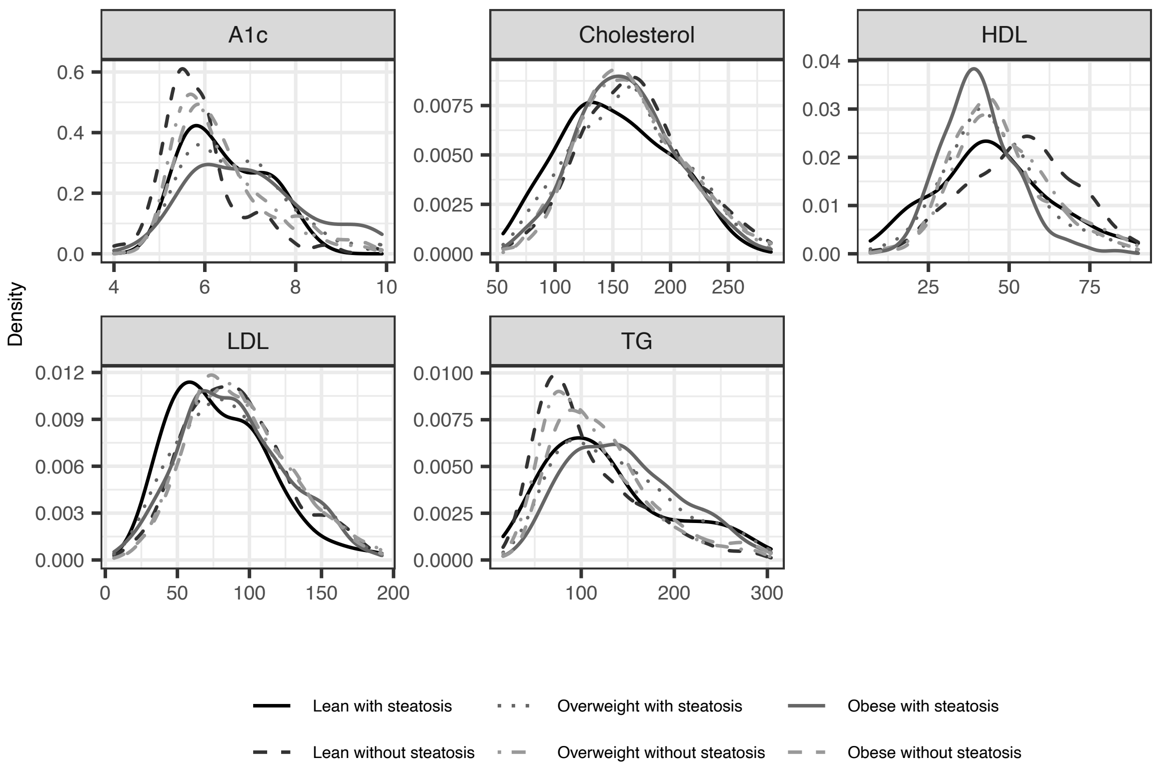

Supplementary Figure S4: Metabolic blood biomarker distributions of the patient cohort split into three BMI categories. Lean patients have 18.5 kg/m^2≤BMI<25 kg/m^2, overweight patients have 25 kg/m^2≤BMI<30 kg/m^2, and obese patients have BMI≥30 kg/m^2. Patients with steatosis have SHAD≥-1 HU or LMA<40 HU. BMI=body mass index, SHAD=spleen-hepatic attenuation difference, LMA=liver mean attenuation, A1c=hemoglobin A1c, HDL=high-density lipoprotein, LDL=low-density lipoprotein, TG=triglycerides.

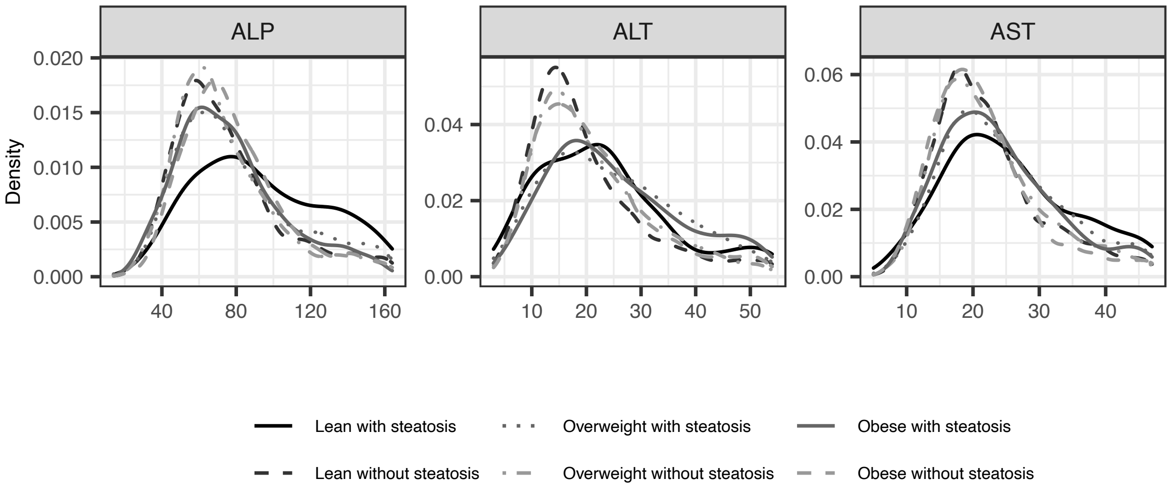

Supplementary Figure S5: Liver function biomarker distributions of the patient groups with obese patients in a third BMI category. Lean patients have 18.5 kg/m^2≤BMI<25 kg/m^2, overweight patients have 25 kg/m^2≤BMI<30 kg/m^2, and obese patients have BMI≥30 kg/m^2. Patients with steatosis have SHAD≥-1 HU or LMA<40 HU. BMI=body mass index, SHAD=spleen-hepatic attenuation difference, LMA=liver mean attenuation, ALP=alkaline phosphatase, ALT=alanine transaminase, AST=aspartate transaminase.

Supplementary Table S10: Biomarker mean values and number of samples over the patient cohort split into three BMI categories.

	Lean with steatosis		Lean without steatosis		Overweight with steatosis		Overweight without steatosis		Obese with steatosis		Obese without steatosis		
	N	Median [IQR]	N	Median [IQR]	N	Median [IQR]	N	Median [IQR]	N	Median [IQR]	N	Median [IQR]	P-value
**A1c (%)**	9	6.6 [1.6]	111	5.7 [0.8]	49	6.6 [1.5]	171	6 [1.25]	125	6.9 [1.9]	197	6.2 [1.3]	<0.0001
**Cholesterol (mg/dL)**	40	156 [66.25]	557	166 [62]	168	162.5 [64.5]	762	162 [63]	365	161 [57]	689	162 [59]	0.54
**HDL (mg/dL)**	36	44.5 [20.5]	494	54 [23]	158	43 [18]	702	47 [20]	341	40 [14]	665	44 [18]	<0.0001
**LDL (mg/dL)**	37	77 [46]	521	86 [49]	153	85 [51]	709	89 [50]	331	87 [51]	651	89 [46]	0.015
**TG (mg/dL)**	42	114.5 [89.25]	624	91.5 [72]	200	130 [92]	812	102 [70]	395	141 [88.5]	736	115 [69]	<0.0001
**ALP (U/L)**	85	96 [60]	1229	70 [35]	376	72 [40.5]	1428	67 [33]	762	72 [36]	1279	73 [32]	<0.0001
**ALT (U/L)**	95	24 [17.5]	1389	17 [12]	410	23 [19]	1657	19 [14]	841	23 [17]	1486	19 [12]	<0.0001
**AST (U/L)**	91	24 [11]	1364	21 [10]	407	23 [12]	1642	21 [11]	840	22 [12]	1450	20 [9]	<0.0001

P-values were calculated using the Kruskal-Wallis test and corrected using Benjamini-Hochberg method for a threshold of $p<0.05$ for statistical significance. Lean patients have 18.5 $kg/m^2 \leq BMI < 25$ kg/m^2, overweight patients have 25 $kg/m^2 \leq BMI < 30$ kg/m^2, and obese patients have $BMI \geq 30$ kg/m^2. Patients with steatosis have SHAD≥-1 HU or LMA<40 HU. BMI=body mass index, SHAD=spleen-hepatic attenuation difference, LMA=liver mean attenuation, IQR=interquartile range, A1c=hemoglobin A1c, ALP=alkaline phosphatase, ALT=alanine transaminase, AST=aspartate transaminase, HDL=high-density lipoprotein, LDL=low-density lipoprotein, TG=triglycerides.

Supplementary Table S11: Z-scores and p-values from Dunn's test for multiple comparisons for biomarkers between the groups of the patient cohort split into three BMI categories.

A. Lean vs obese patients

	Lean with steatosis vs Obese with steatosis		Lean without steatosis vs Obese with steatosis		Lean with steatosis vs Obese without steatosis		Lean without steatosis vs Obese without steatosis	
	Z	P	Z	P	Z	P	Z	P
A1c (%)	-1.43	0.21	-7.09	**<0.0001**	0.21	0.87	-4.37	**<0.0001**
Cholesterol (mg/dL)	-1.82	0.11	1.90	0.094	-2.55	**0.021**	0.93	0.42
HDL (mg/dL)	2.43	**0.029**	12.05	**<0.0001**	-0.51	0.66	7.88	**<0.0001**
LDL (mg/dL)	-2.24	**0.043**	0.55	0.64	-3.28	**2.7E-3**	-1.24	0.28
TG (mg/dL)	-3.22	**3.2E-3**	-11.20	**<0.0001**	-0.28	0.82	-7.08	**<0.0001**
ALP (U/L)	5.96	**<0.0001**	-1.915	0.091	5.23	**<0.0001**	-4.06	**1.5E-4**
ALT (U/L)	-2.35	**0.036**	-10.89	**<0.0001**	2.26	**0.042**	-3.71	**6.1E-4**
AST (U/L)	2.16	0.052	-4.14	**1.1E-4**	5.92	**<0.0001**	2.28	**0.041**

B. Overweight vs obese patients

	Overweight with steatosis vs Obese with steatosis		Overweight without steatosis vs Obese with steatosis		Overweight with steatosis vs Obese without steatosis		Overweight without steatosis vs Obese without steatosis	
	Z	P	Z	P	Z	P	Z	P
A1c (%)	-1.18	0.30	-4.66	**<0.0001**	1.33	0.25	-1.30	**0.26**
Cholesterol (mg/dL)	0.17	0.88	0.94	0.42	-0.70	0.55	-0.31	0.81
HDL (mg/dL)	3.06	**4.9E-3**	8.32	**<0.0001**	-1.00	0.39	3.07	**4.9E-3**
LDL (mg/dL)	-0.13	0.90	1.55	0.17	-1.41	0.22	-0.19	0.88
TG (mg/dL)	-1.79	0.11	-9.18	**<0.0001**	2.29	**0.040**	-4.41	**<0.0001**
ALP (U/L)	1.30	0.26	-2.82	**0.010**	0.09	0.93	-5.26	**<0.0001**
ALT (U/L)	-0.60	0.62	-8.45	**<0.0001**	5.49	**<0.0001**	-0.42	0.72
AST (U/L)	1.40	0.22	-3.65	**7.4E-4**	6.38	**<0.0001**	3.27	**2.7E-3**

P-values were corrected using the Benjamini-Hochberg method for a threshold of $p<0.05$ for statistical significance. Lean patients have 18.5 $kg/m^2 \leq BMI < 25\ kg/m^2$, overweight patients have 25 $kg/m^2 \leq BMI < 30\ kg/m^2$, and obese patients have $BMI \geq 30\ kg/m^2$. Patients with steatosis have SHAD≥-1 HU or LMA<40 HU. BMI=body mass index, SHAD=spleen-hepatic attenuation difference, LMA=liver mean attenuation, A1c=hemoglobin A1c, ALP=alkaline phosphatase, ALT=alanine transaminase, AST=aspartate transaminase, HDL=high-density lipoprotein, LDL=low-density lipoprotein, TG=triglycerides.

Supplementary Table S12: FIB-4 scores for lean, overweight, and obese patients with steatosis.

	Lean with steatosis	Overweight with steatosis	Obese with steatosis
N	124	222	461
Severe fibrosis (n (%))	18 (14.5%)	31 (14.0%)	25 (5.4%)
Median [IQR]	1.52 [1.24]	1.52 [1.22]	1.33 [0.94]
Severe fibrosis % vs obese with steatosis (p-value)	1.2E-3	2.5E-4	-
Distribution vs obese with steatosis (p-value)	0.082	2.1E-4	-

P-values were calculated using the chi-square test and the Wilcoxon test in comparing proportion of patients with severe fibrosis and FIB-4 distributions, respectively. A FIB-4 score of FIB-4≥3.25 was considered to indicate severe fibrosis. Lean patients have 18.5 $kg/m^2 \leq BMI < 25\ kg/m^2$, overweight patients have 25 $kg/m^2 \leq BMI < 30\ kg/m^2$, and obese patients have $BMI \geq 30\ kg/m^2$. Patients with steatosis have SHAD≥-1 HU or LMA<40 HU. BMI=body mass index, SHAD=spleen-hepatic attenuation difference, LMA=liver mean attenuation.

Supplementary Table S13: Top 200 phenotypes by significance of the association with SHAD in lean patients.

Top 200 Phenotypes by Significance of Association with *SHADxLEAN*<br>Corrected statistical significance threshold: p<7.52E-5		
Phenotype	P-Value	Coefficient (SHAD*LEAN)
**Disorders of fluid, electrolyte, and acid-base balance**	**4.06E-09**	**0.045481**
**Other anemias**	**1.30E-08**	**0.044958**
**Electrolyte imbalance**	**1.05E-07**	**0.043696**
**Septicemia**	**2.05E-06**	**0.058455**
**Congestive heart failure; non-hypertensive**	**3.90E-06**	**0.03562**
**Congestive heart failure (CHF) not otherwise specified (NOS)**	**6.83E-06**	**0.03985**
**Renal failure**	**9.02E-06**	**0.035228**
**Thrombocytopenia**	**2.52E-05**	**0.0477**
**Purpura and other hemorrhagic conditions**	**4.01E-05**	**0.045372**
**Chronic renal failure [CKD]**	**6.96E-05**	**0.03399**
Hypopotassemia	9.82E-05	0.049698
Fluid overload	0.000126	0.067983
Encounter for long-term (current) use of anticoagulants	0.000155	0.033156
Hypovolemia	0.000161	0.04851
Anemia in chronic kidney disease	0.000161	0.057442
Pulmonary heart disease	0.000191	0.033274
Kidney replaced by transplantation	0.000309	0.045704
Complications of transplantation and reattached limbs	0.000435	0.038337
Fracture of vertebral column without mention of spinal cord injury	0.000463	0.076972
Heart failure with reduced ejection fraction (EF) [Systolic or combined heart failure]	0.000746	0.032863
Heart valve replacement	0.00075	0.041141
Sepsis	0.000802	0.046103
Respiratory failure, insufficiency, arrest	0.000861	0.033955
Hypoosmolality and/or hyponatremia	0.00088	0.043656
Sepsis and systemic inflammatory response syndrome (SIRS)	0.00101	0.043125
Bacterial infection NOS	0.00103	0.032928
Valvular heart disease/ heart chambers	0.00108	0.076333
Acid-base balance disorder	0.00108	0.04579
Back pain	0.00113	-0.028933
Cardiomyopathy	0.00113	0.029582
Pleurisy; pleural effusion	0.00136	0.039658
Chronic pulmonary heart disease	0.00137	0.031425
Hypertensive chronic kidney disease	0.00141	0.037941
End stage renal disease	0.00156	0.036777
Anemia of chronic disease	0.00163	0.04084
Acute renal failure	0.0018	0.028953
Fever of unknown origin	0.0023	0.034794
Proteinuria	0.00252	0.038522
Rheumatic disease of the heart valves	0.00274	0.032513
Dry eyes	0.00308	-0.066066
Primary/intrinsic cardiomyopathies	0.00341	0.027918
Cardiac defibrillator in situ	0.00388	0.033243
Cardiac pacemaker/device in situ	0.00388	0.028451

Hyperpotassemia	0.00408	0.032903
Atrial fibrillation and flutter	0.00419	0.025915
Hypertensive heart and/or renal disease	0.00468	0.030933
Atrial fibrillation	0.00473	0.026133
Paroxysmal ventricular tachycardia	0.00501	0.032653
Disturbance of skin sensation	0.0053	-0.038505
Calculus of kidney	0.00572	-0.035374
Hypersomnia	0.00735	-0.10259
Dependence on respirator [Ventilator] or supplemental oxygen	0.00744	0.039162
Hypertension	0.00756	0.021659
Primary pulmonary hypertension	0.00783	0.044724
Nephritis; nephrosis; renal sclerosis	0.00815	0.043332
Essential hypertension	0.00897	0.021376
Heart valve disorders	0.0107	0.020229
Abnormal weight gain	0.0107	-0.087895
Premature beats	0.0117	0.033746
Pneumonia	0.0122	0.022031
Coagulation defects	0.0126	0.036132
Hypotension NOS	0.0129	0.042171
Thyrotoxicosis with or without goiter	0.0135	0.04548
Cardiac dysrhythmias	0.016	0.017485
Neuroendocrine tumors	0.0169	0.053549
Endocarditis	0.017	0.05271
Iron deficiency anemias	0.0187	0.02672
Nonrheumatic aortic valve disorders	0.0198	0.024484
Acidosis	0.0211	0.037076
Heart failure with preserved EF [Diastolic heart failure]	0.0213	0.028398
Other immunological findings	0.0213	0.037678
Diseases of white blood cells	0.0237	0.029619
Tinnitus	0.0242	-0.044518
Other conditions of brain	0.0242	0.045985
Paroxysmal tachycardia, unspecified	0.0244	0.023074
Epilepsy, recurrent seizures, convulsions	0.025	0.03447
Nonrheumatic mitral valve disorders	0.0251	0.021785
Acute pulmonary heart disease	0.0258	0.035448
Gross hematuria	0.0261	-0.051221
Gout	0.0269	0.026144
Inflammatory diseases of prostate	0.0271	-0.049684
Disorders involving the immune mechanism	0.0273	0.023978
Congenital anomalies of urinary system	0.0277	0.03505
Hemoptysis	0.0279	-0.053312
Iron deficiency anemias, unspecified or not due to blood loss	0.028	0.025883
Respiratory failure	0.0283	0.030555
Cardiac congenital anomalies	0.0283	0.034422
Benign mammary dysplasia	0.0285	-0.065034
Carditis	0.0291	0.029362
Pulmonary embolism and infarction, acute	0.0297	0.034833
Elevated white blood cell count	0.0301	0.031637
Sleep disorders	0.0304	-0.021502

Seborrheic keratosis	0.0306	-0.028576
Urinary calculus	0.0311	-0.024654
Mood disorders	0.0315	0.018304
Pain in joint	0.0317	-0.016464
Ovarian cyst	0.0324	-0.048727
Ischemic Heart Disease	0.0326	0.016145
Convulsions	0.0332	0.036626
Hyperlipidemia	0.0348	0.015303
Poisoning by primarily systemic agents	0.035	0.034371
Chronic Kidney Disease, Stage III	0.035	0.022856
Insomnia	0.0352	-0.023514
Viral infection	0.0355	0.025528
Depression	0.0357	0.018163
Abnormal glucose	0.0357	-0.021107
Disorders of lipoid metabolism	0.0373	0.015081
Antineoplastic and immunosuppressive drugs causing adverse effects	0.0378	0.033889
Other disorders of the kidney and ureters	0.0386	0.020981
Gout and other crystal arthropathies	0.041	0.023614
Open wounds of extremities	0.0427	0.042626
Pain	0.043	-0.025871
Cystic kidney disease	0.0442	0.03365
Sleep related movement disorders	0.0447	-0.048012
Mitral valve disease	0.0448	0.032851
Genitourinary congenital anomalies	0.0452	0.031165
Chronic ulcer of skin	0.0455	0.028255
Complications of cardiac/vascular device, implant, and graft	0.0456	0.031638
Nontoxic multinodular goiter	0.0458	-0.034238
Solitary pulmonary nodule	0.0463	-0.018979
Glomerulonephritis	0.0476	0.046994
Bacterial pneumonia	0.0478	0.025969
Neuralgia, neuritis, and radiculitis NOS	0.0479	-0.052165
Other disorders of intestine	0.0479	-0.043469
Voice disturbance	0.0485	-0.031141
Fracture of ankle and foot	0.0493	-0.063211
Secondary malignant neoplasm of liver	0.0509	0.038925
Frequency of urination and polyuria	0.0515	-0.023115
Cardiac pacemaker in situ	0.0525	0.025735
Cardiomegaly	0.0535	0.044491
Cancer of prostate	0.0543	-0.032709
Testicular dysfunction	0.0552	-0.050713
Erythematous conditions	0.0573	0.024264
Heart transplant/surgery	0.0591	0.024623
Osteopenia or other disorder of bone and cartilage	0.0618	-0.020391
Atrial flutter	0.0621	0.025102
Testicular hypofunction	0.0634	-0.049161
Cancer of urinary organs (incl. kidney and bladder)	0.0637	-0.021921
Coronary atherosclerosis	0.0642	0.014591
Other disorders of synovium, tendon, and bursa	0.0642	-0.027029
Inflammation of eyelids	0.0648	-0.035525

Other diseases of blood and blood-forming organs	0.0694	0.048272
Other abnormal glucose	0.0702	-0.019629
Aplastic anemia	0.0705	0.037685
Cellulitis and abscess of leg, except foot	0.0709	0.043919
Other venous embolism and thrombosis	0.0709	0.018451
Cardiac conduction disorders	0.0715	0.015526
Myalgia and myositis unspecified	0.0717	-0.024081
Bacteremia	0.0719	0.037358
Mitral valve stenosis and aortic valve stenosis	0.0738	0.041532
Atrioventricular block, complete	0.0755	0.031886
Disorders of function of stomach	0.0773	0.025422
Other disorders of circulatory system	0.0783	0.024674
Postmenopausal bleeding	0.079	-0.052631
Colon cancer	0.0807	0.031621
Chronic ulcer of leg or foot	0.0814	0.035148
Other symptoms of respiratory system	0.0815	-0.012191
Restless legs syndrome	0.0823	-0.048332
Sciatica	0.0826	-0.028161
Secondary/extrinsic cardiomyopathies	0.0838	0.026366
Other hypertensive complications	0.0861	0.024991
Chronic venous insufficiency [CVI]	0.0862	0.043875
Osteoporosis NOS	0.0868	0.021841
Hematuria	0.0869	-0.018765
Prostatitis	0.0871	-0.043723
Extrapyramidal disease and abnormal movement disorders	0.0885	0.038518
Mixed hyperlipidemia	0.0916	0.015291
Cerebrovascular disease	0.0928	0.014544
Diseases of the larynx and vocal cords	0.093	-0.023286
Genital prolapse	0.0933	-0.039289
Swelling, mass, or lump in head and neck [Space-occupying lesion, intracranial NOS]	0.0934	-0.032623
Other dyspnea	0.0944	-0.015504
Chronic pain	0.0958	-0.024975
Disorders of calcium/phosphorus metabolism	0.0958	0.021448
Benign neoplasm of other parts of digestive system	0.0961	-0.048084
Hyperplasia of prostate	0.097	-0.021727
Squamous cell carcinoma	0.098	0.030342
Renal dialysis	0.0994	0.024896
Other disorders of bladder	0.0997	-0.024573
Synovitis and tenosynovitis	0.1	-0.034547
Colorectal cancer	0.102	0.027754
Spasm of muscle	0.103	-0.049244
Encounter for long-term (current) use of aspirin	0.103	0.01925
Disturbances of sensation of smell and taste	0.103	-0.041397
Shock	0.105	0.032134
Deep vein thrombosis [DVT]	0.107	0.020328
Shortness of breath	0.108	-0.013462
Spondylosis without myelopathy	0.109	-0.025208
Retention of urine	0.111	-0.023392
Sinoatrial node dysfunction (Bradycardia)	0.113	0.025873

Other disorders of male genital organs	0.114	-0.041656
Peripheral vascular disease	0.114	0.016112
Gastroparesis	0.114	0.028056
Pruritus and related conditions	0.115	0.024166
Other disorders of thyroid	0.117	-0.034678
Fasciitis	0.12	-0.05833
Asthma	0.12	0.015466
Abnormal findings on mammogram or breast exam	0.12	-0.023604
Inflammatory and toxic neuropathy	0.121	0.026301
Cancer of larynx	0.121	-0.038571

Coefficients and p-values are from the *SHAD x LEAN* statistical interaction term of the logistic regression model controlling for age, sex, and race. LEAN is a binary characteristic indicating that a patient has 18.5 $kg/m^2 \leq BMI < 25\ kg/m^2$. A p-value of $p<7.52x10^{-5}$ indicates significance with the Bonferroni multiple comparison correction. BMI=body mass index, SHAD=spleen-hepatic attenuation difference.

Supplementary Table S14: Top 200 phenotypes by significance of the association with interaction between PDFF and lean characteristic in the UK Biobank cohort

Phenotype	P-Value	Coefficient (PDFF*LEAN)	Cases
Diaphragmatic hernia	4.48E-05	0.051232	1735
Heartburn	0.000337	0.10293	162
Type 1 diabetes with ophthalmic manifestations	0.00073	2.0105	11
Abdominal hernia	0.00218	0.037578	2065
Reflux esophagitis	0.00241	0.056659	707
Diseases of the larynx and vocal cords	0.00246	0.094087	140
Altered mental status	0.00327	-1.914	43
Esophagitis, GERD and related diseases	0.00439	0.039193	1486
Alcoholism	0.00456	0.056869	621
Voice disturbance	0.0058	0.10014	73
Perforation of tympanic membrane	0.00604	0.11638	68
Prurigo and Lichen	0.0063	0.1172	78
Varicose veins	0.00657	0.15865	76
Myopathy	0.0066	0.20438	14
Diseases of esophagus	0.00963	0.034402	1670
Other disorders of stomach and duodenum	0.00995	-0.39682	164
Duodenitis	0.0116	0.048359	527
Bacterial infection NOS	0.0118	0.056806	388
E. coli	0.0127	0.10184	66
Cellulitis and abscess of face/neck	0.0164	0.16338	38
Tobacco use disorder	0.0165	0.04191	732
Asthma	0.0225	0.03016	1763
Inguinal hernia	0.0227	0.043347	1254
Benign neoplasm of brain and other parts of nervous system	0.0231	0.15477	17
Poisoning by psychotropic agents	0.0247	0.10682	76
Cholelithiasis	0.0251	0.048569	535
Benign neoplasm of brain, cranial nerves, meninges	0.0299	0.15018	15
Heart valve replaced	0.0306	-3.5732	5
Other and unspecified disorders of the nervous system	0.0306	0.14956	13
Other disorders of eye	0.0311	0.19922	25
Neuralgia, neuritis, and radiculitis NOS	0.0336	-2.0881	14
Shortness of breath	0.0338	0.058225	260
Other disorders of cervical region	0.034	0.15595	15
Disorders of the autonomic nervous system	0.0341	-4.5237	5
Hemorrhoids	0.0346	-0.037251	1958
Otitis externa	0.0351	0.18203	19
Large cell lymphoma	0.036	-1.5222	23
Hypothyroidism NOS	0.0381	0.036461	792
Vitamin B-complex deficiencies	0.0382	0.16458	39
Muscular dystrophies and other myopathies	0.04	0.23888	10

Systemic sclerosis	0.0401	0.24523	15
Jaundice (not of newborn)	0.0401	0.22676	32
Periodontitis (acute or chronic)	0.0425	0.099396	75
Intervertebral disc disorder with myelopathy	0.0425	0.14762	14
Diffuse diseases of connective tissue	0.0443	0.15063	21
Calculus of bile duct	0.0454	0.071901	120
Congenital anomalies of genital organs	0.0456	0.11528	61
Urinary incontinence	0.0458	0.037805	596
Benign neoplasm of other parts of digestive system	0.0461	0.05487	350
Gastritis and duodenitis	0.0474	0.032031	1028
Blood in stool	0.0475	0.067106	126
Neurological disorders	0.049	-0.42826	96
Aneurysm and dissection of heart	0.0492	-0.69182	31
Other disorders of prostate	0.0501	0.10549	107
Contusion	0.0511	0.11367	71
Other specified nonpsychotic and/or transient mental disorders	0.0517	0.18036	14
Hypothyroidism	0.0536	0.033886	820
Arterial embolism and thrombosis	0.0559	0.12032	15
Secondary malignant neoplasm of liver	0.0561	-3.7146	4
Anemia of chronic disease	0.0594	1.2974	11
Myasthenia gravis	0.0613	0.1637	10
Postmenopausal atrophic vaginitis	0.0616	-0.33354	60
Vaginal enterocele, congenital or acquired	0.0629	0.11625	44
Acute renal failure	0.0635	-0.46613	74
Cholelithiasis with other cholecystitis	0.0643	0.048852	321
Hemiplegia	0.0645	0.10575	44
Other symptoms involving abdomen and pelvis	0.0661	-0.16503	188
Early or threatened labor; hemorrhage in early pregnancy	0.0661	0.056259	230
Fracture of lower limb	0.0663	0.1227	36
Other allied disorders of spine	0.0665	-1.9526	12
Anxiety disorders	0.0669	0.045997	276
Genital prolapse	0.0681	0.036808	619
Other disorders of circulatory system	0.0682	-3.4235	8
Benign neoplasm of adrenal gland	0.069	-8.0005	9
Atrophy of female genital tract	0.0691	0.16014	25
Otitis media	0.0714	-0.42483	69
Chronic periodontitis	0.0726	0.074726	105
Wegener's granulomatosis	0.0742	0.13454	12
GERD	0.076	0.030499	854
Duodenal ulcer	0.0776	0.059392	184
Fracture of patella	0.0837	0.26428	11
Periapical abscess	0.0838	0.073365	93
Depression	0.0861	0.033167	528
Other acquired musculoskeletal deformity	0.0864	-0.36498	63
Major depressive disorder	0.0871	0.033048	525

Noninflammatory disorders of vulva and perineum	0.0878	-0.18917	121
Pneumonia	0.0886	0.075735	119
Streptococcus infection	0.0886	0.080708	68
Superficial injury without mention of infection	0.0891	-0.13677	213
Disorders of parathyroid gland	0.0894	-0.6885	43
Pain in joint	0.091	-0.23864	109
Iron deficiency anemias, unspecified or not due to blood loss	0.0919	-0.069805	419
Prolapse of vaginal walls	0.0946	0.034786	404
Aphakia and other disorders of lens	0.0948	0.081628	102
Excessive vomiting in pregnancy	0.0948	-0.70848	28
Anemia in neoplastic disease	0.0951	1.6464	7
Immunity deficiency	0.0956	-2.3029	9
Abnormal function study of cardiovascular system	0.0995	0.12435	30
Anxiety disorder	0.1	0.042883	287
Acquired spondylolisthesis	0.1	-0.43743	40
Mood disorders	0.102	0.03161	554
Other unspecified back disorders	0.102	0.12021	15
Hypertrophy of female genital organs	0.103	-0.23049	133
Ventricular fibrillation and flutter	0.103	0.34406	10
Hyperparathyroidism	0.104	-0.68521	40
Delirium due to conditions classified elsewhere	0.105	0.22892	7
Diseases of lips	0.107	0.082329	61
Sialolithiasis	0.109	0.12898	34
Other cerebral degenerations	0.109	-1.6318	9
Skull and face fracture and other intercranial injury	0.109	-0.21153	99
Acidosis	0.112	-0.82544	16
Cerebral degeneration, unspecified	0.113	-4.5198	3
Simple and unspecified goiter	0.114	0.14505	39
Iron deficiency anemias	0.114	-0.062513	441
Dizziness and giddiness (Light-headedness and vertigo)	0.115	0.050853	260
Lupus (localized and systemic)	0.115	-1.0505	21
Anomalies of pupillary function	0.117	0.35674	12
Vitamin deficiency	0.118	0.11153	59
Cellulitis and abscess of oral soft tissues	0.119	0.13128	14
Benign neoplasm of pituitary gland and craniopharyngeal duct (pouch)	0.122	0.50769	11
Umbilical hernia	0.124	-0.16511	211
Irritable Bowel Syndrome	0.124	0.033723	377
Meningitis	0.126	0.096101	30
Disorders of iron metabolism	0.127	-1.3067	35
Other peripheral nerve disorders	0.128	-0.043909	793
Aneurysm of artery of lower extremity	0.128	-4.5196	5
Alcoholic liver damage	0.128	-2.2268	9
Acute tonsillitis	0.129	0.07716	53
Postoperative infection	0.129	0.052223	145
Post inflammatory pulmonary fibrosis	0.131	-1.1014	22

Coronary atherosclerosis	0.131	0.032035	952
Symptoms involving digestive system	0.134	-0.033768	1088
Diseases of the jaws	0.134	-0.70652	22
Benign neoplasm of skin	0.135	0.030859	769
Infection/inflammation of internal prosthetic device; implant; and graft	0.135	0.12013	39
Other diseases of lung	0.138	-0.92259	21
Calculus of ureter	0.139	0.043828	199
Malaise and fatigue	0.139	-0.38224	45
Anomalies of tooth position/malocclusion	0.141	0.071295	33
Ovarian cyst	0.141	-0.05455	416
Known or suspected fetal abnormality affecting management of mother	0.141	-0.056584	435
Uterine/Uterovaginal prolapse	0.142	0.037838	386
Anticoagulants causing adverse effects	0.142	1.0128	5
Lymphadenitis	0.144	-0.16305	124
Encephalitis	0.144	0.19578	26
Personal history of allergy to medicinal agents	0.145	-0.12275	224
Fracture of upper limb	0.145	0.24585	16
Other anemias	0.146	-0.050019	516
Suicide or self-inflicted injury	0.147	-0.55827	43
Hallux valgus (Bunion)	0.148	0.047852	357
Cancer of tongue	0.148	-4.1584	13
Hemoptysis	0.148	-0.13511	93
Diplopia and disorders of binocular vision	0.15	-0.45075	32
Transient cerebral ischemia	0.15	-0.23763	101
Retinal detachments and defects	0.151	0.083249	152
Subjective visual disturbances	0.152	0.10697	34
Congenital anomalies of female genital organs	0.152	0.081943	50
Hemorrhage or hematoma complicating a procedure	0.152	0.038912	261
Other tests	0.153	0.036046	558
Celiac disease	0.153	-0.12084	125
Hyperhidrosis	0.154	0.090038	31
Fluid overload	0.155	-4.0096	6
Memory loss	0.155	-0.44544	34
Inflammation of eyelids	0.156	-0.069372	215
Abdominal pain	0.156	-0.020025	2221
Intestinal obstruction without mention of hernia	0.158	0.095538	42
Diseases and other conditions of the tongue	0.158	-0.18381	89
Vascular disorders of penis	0.159	0.089537	48
Gingival and periodontal diseases	0.161	0.060752	97
Dystrophy of female genital tract	0.163	-1.0781	19
Cystic mastopathy	0.163	-0.21113	75
Chronic pulmonary heart disease	0.163	0.16223	8
Venous/cerebrovascular complications embolism in pregnancy and the puerperium	0.164	-0.45102	29
Folate-deficiency anemia	0.164	-1.1978	8
Chronic fatigue syndrome	0.164	-0.3545	43

Symptomatic menopause	0.166	-1.4114	15
Pallor and flushing	0.166	0.23768	9
Other symptoms/disorders or the urinary system	0.166	0.02467	888
Myalgia and myositis unspecified	0.17	0.07076	41
Respiratory abnormalities	0.17	-0.70161	30
Hyperemesis gravidarum	0.173	-0.63101	21
Type 1 diabetes	0.173	-0.31604	81
First degree AV block	0.174	-0.82959	32
Urinary tract infection	0.174	0.031765	560
Secondary malignancy of bone	0.175	-1.61	10
Proliferative glomerulonephritis	0.175	2.3964	3
Precordial pain	0.176	-0.10439	231
Urethritis and urethral syndrome	0.177	0.36775	8
Neural tube defects	0.178	-3.8821	4
Nonspecific abnormal findings in stool contents	0.18	-0.11506	125
Hodgkin's disease	0.18	0.12707	10
Psychogenic disorder	0.181	0.11294	29
Pelvic inflammatory disease (PID)	0.181	0.073136	26
Bacterial pneumonia	0.181	0.093129	29
Impaction of intestine	0.183	1.3159	6
Hemangioma and lymphangioma, any site	0.184	-0.12429	125
Nodular lymphoma	0.185	-0.77983	20
Malignant and unknown neoplasms of brain and nervous system	0.186	0.2966	7
Other mental disorder	0.188	0.02123	1472
Cerebral ischemia	0.188	-0.18782	118

Coefficients and p-values are from the *PDFF x LEAN* statistical interaction term of the logistic regression model controlling for age, sex, and race. LEAN is a binary characteristic indicating that a patient has 18.5 kg/m^2≤BMI<25 kg/m^2. BMI=body mass index, PDFF=proton density fat fraction.

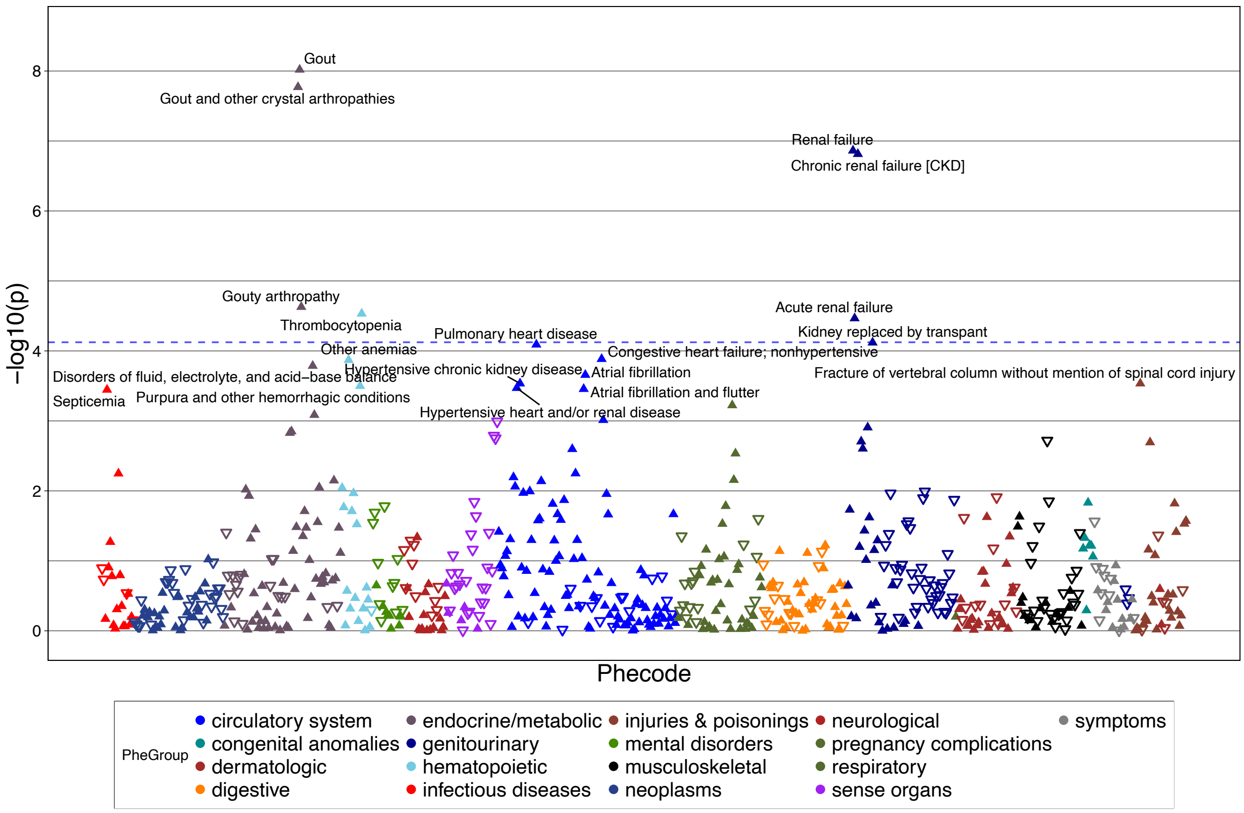

Supplementary Figure S6: Phenome-Wide Association Study of the statistical interaction between the continuous characteristics SHAD and BMI (*SHAD x BMI*). Each coded phenotype is shown as a triangle and further grouped by color as indicated in the legend at the bottom. Upward pointing triangles indicate a positive association with *SHAD x BMI*, while downward pointing triangles indicate a negative association. The blue dashed horizontal line on the graph indicates the level of statistical significance with Bonferroni multiple-comparison correction ($p<7.5x10^{-5}$). Phenotypes above the line are those that show a statistically significant association with *SHAD x BMI*. The seven statistically significant phenotypes in order of statistical significance are the following: gout, gout and other crystal arthropathies, renal failure, chronic renal failure (CKD), gouty arthropathy, thrombocytopenia, acute renal failure. BMI=body mass index, SHAD=spleen-hepatic attenuation difference.

Supplementary Table S15: ICD-9 codes used for exclusion.

Condition	ICD-9 codes
Alcohol-related conditions	317, 317.1, 317.11
ESLD complications	530.2, 567, 571.51, 571.8, 572
Bariatric surgery	539
Viral hepatitis	070, 070.1, 070.2, 070.3
Cachexia	260.1
Cancer	145, 145.1, 145.2, 145.3, 145.4,145.5, 149, 149.1, 149.2, 149.3, 149.4, 149.9, 150, 151, 153,, 153.2, 153.3, 155, 155.1, 157, 159, 159.2, 159.3, 159.4, 164, 165, 165.1, 170, 170.1, 170.2, 172, 172.11, 172.2, 172.21, 172.22, 172.3, 174, 174.1, 174.11, 174.2, 180, 180.1, 180.3, 182, 184, 184.1, 184.2, 185, 187, 187.1, 187.2, 189.1, 189.2, 189.21, 189.4, 190, 191.1, 191.11, 193, 194, 195, 195.1, 198, 198.1, 198.2, 198.3, 198.4, 198.5, 198.6, 198.7, 201, 202, 202.2, 202.21, 202.22, 202.23, 202.24, 204, 204.1, 204.11, 204.12, 204.2, 204.21, 204.22, 204.3, 230, 851

ESLD=end-stage liver disease.

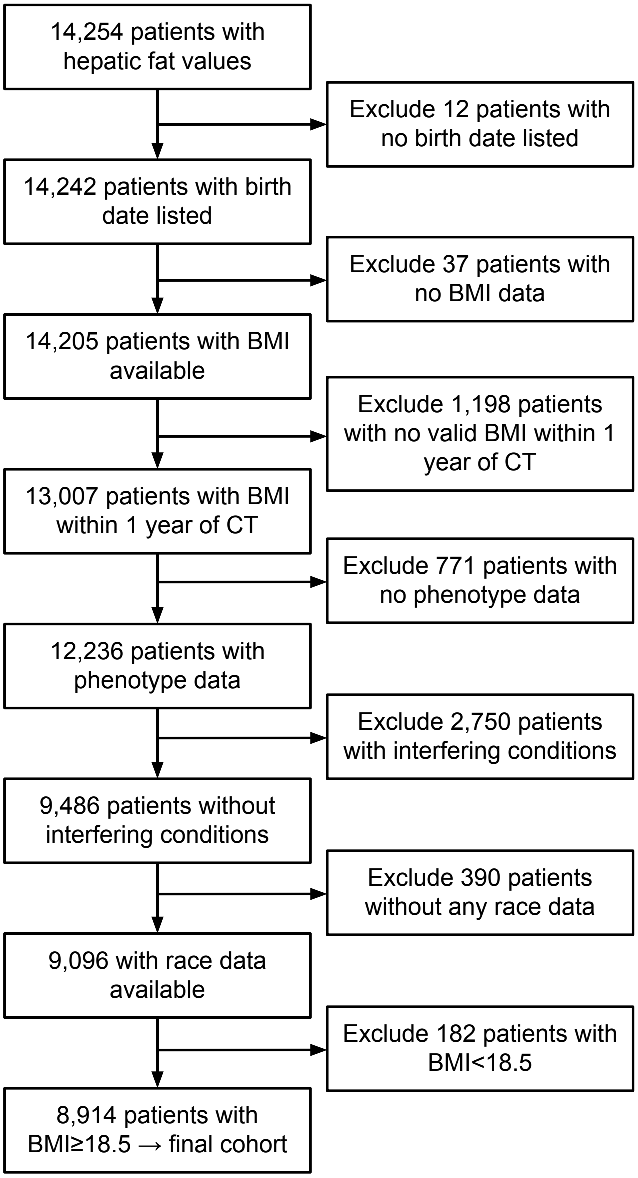

Supplementary Figure S7: Flow chart illustrating the number of patients in the study cohort out of the total number with an available SHAD value after applying each exclusion criterion. Interfering conditions considered for this study were end-stage liver disease complications, alcohol use disorder, viral hepatitis, bariatric surgery, cachexia, and cancer.

Penn Medicine BioBank Banner Author List and Contribution Statements

PMBB Leadership Team
Daniel J. Rader, M.D., Marylyn D. Ritchie, Ph.D.

Contribution: All authors contributed to securing funding, study design and oversight. All authors reviewed the final version of the manuscript.

Patient Recruitment and Regulatory Oversight
JoEllen Weaver, Nawar Naseer, Ph.D., M.P.H., Afiya Poindexter, Khadijah Hu-Sain, Yi-An Ko, Ph.D.

Contributions: JW manages patient recruitment and regulatory oversight of study. NN manages participant engagement, assists with regulatory oversight, and researcher access. AP, KH, YK perform recruitment and enrollment of study participants.

Lab Operations
JoEllen Weaver, Meghan Livingstone, Fred Vadivieso, Stephanie DerOhannessian, Teo Tran, Julia Stephanowski, Monica Zielinski, Ned Haubein, Joseph Dunn

Contribution: JW, ML, FV, SD conduct oversight of lab operations. ML, FV, AK, SD, TT, JS, MZ perform sample processing. NH, JD are responsible for sample tracking and the laboratory information management system.

Clinical Informatics
Anurag Verma, Ph.D., Colleen Morse Kripke, M.S. DPT, MSA, Marjorie Risman, M.S., Renae Judy, B.S.

Contribution: All authors contributed to the development and validation of clinical phenotypes used to identify study subjects and (when applicable) controls.

Genome Informatics
Anurag Verma Ph.D., Shefali S. Verma, Ph.D., Yuki Bradford, M.S., Scott Dudek, M.S., Theodore Drivas, M.D., Ph.D.

Contribution: A.V., S.S.V. are responsible for the analysis, design, and infrastructure needed to quality control genotype and exome data. Y.B. performs the analysis. T.D. and A.V. provides variant and gene annotations and their functional interpretation of variants.
